# Synergistic Intra‐ and Inter‐Nanozyme Electron Transfer through Interfacial Assembly for Enhanced Multi‐Enzyme Activity

**DOI:** 10.1002/advs.202524274

**Published:** 2026-01-28

**Authors:** Kun Lu, Jizi Liu, Xiaoyang Zhu, Yu Mao, Hongliang He, Pingqiang Cai, Yan Li, Ning Gu

**Affiliations:** ^1^ Jiangsu Key Laboratory for Biomaterials and Devices School of Biological Science and Medical Engineering Southeast University Nanjing P. R. China; ^2^ Jiangsu Key Laboratory for Cardiovascular Information and Health Engineering Medicine Nanjing Research Center for Biomedical Electron Microscopy, Institute of Clinical Medicine, Nanjing Drum Tower Hospital, Medical School, Nanjing University Nanjing P. R. China; ^3^ Jiangsu Key Laboratory of Molecular Medicine Medical School Nanjing University Nanjing P. R. China

**Keywords:** composite nanozyme, electron transfer, molybdenum disulfide, prussian blue, redox

## Abstract

Prussian Blue composite nanozymes (PB C‐NZs) have been extensively employed in various biomedical applications. However, due to the complexity of its multi‐enzyme activities and structural components, designing material composition and processes, optimizing specific enzymatic properties of materials, and exploring the scientific mechanisms involved remain important challenges. Given the equal importance of electron transfer optimization and intrinsic redox properties, we propose an interfacial assembly strategy that leverages electron transfer and energy band structure, allowing for the synergistic interactions between internal and interfacial electrons of the nanocomposites (MoS_2_/PB) and thereby enhancing the enzyme‐like activity. In the MoS_2_/PB system, a distinct inter‐nanozyme electron transfer is operative, facilitating directional electron transport from MoS_2_ to PB, thereby enhancing catalytic activity. Concurrently, band modulation effects induced by the interaction between MoS_2_ and PB effectively enhance the reductase‐like catalytic activity. Notably, the expression of multiple enzyme activities can be enriched through band regulation. The comprehensive enzymatic activity tests demonstrated that MoS_2_/PB exhibits enhanced multiple‐enzyme activities, including catalase, peroxidase, superoxide dismutase, glutathione peroxidase, S‐nitrosoglutathione reductase, and nitrite reductase. This study introduces a novel design concept for composite nanozymes based on electron transfer modulation, providing valuable insights and guidance for the development of high‐performance nanozymes.

## Graphical abstract

A novel Prussian blue‐based composite nanoenzymes (PB C‐NZs)‐MoS_2_/PB, was designed based on the electron transfer mechanism and transport pathway. The structure uses MoS_2_ as the substrate, with PB grown on its surface through a surface assembly method. The relatively high energy band position of MoS_2_ not only facilitates intrinsic electron transfer during the catalytic process but also promotes electron transfer from the interface to PB. The synergistic electron transfer within and between the nanoenzymes enhances the catalytic performance of MoS_2_/PB, while the incorporation of MoS_2_ enriches the multi‐enzyme activity expression of PB.

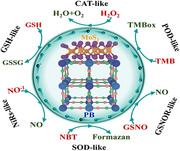

## 1 Introduction

Natural enzymes play a crucial role in biological systems and have been widely applied in the biomedical field. However, their poor chemical stability, limited application methods, and high production costs seriously hinder their practical implementation. Nanozymes, as emerging and efficient biocatalysts, exhibit the capability to regulate the levels of reactive oxygen species (ROS) in living organisms, thereby contributing to the homeostasis of the biological environments and demonstrating immense potential in biomedical applications.[1] Among them, Prussian Blue nanozymes (PBNZs), have attracted increasing attention in recent years and have been widely used in many diagnostic and therapeutic fields.[2, 3, 4] Their exceptional performance across diverse biological applications is mainly attributed to their unique structural units and excellent electron transfer efficiency.[5, 6, 7] Meanwhile, the development of PB C‐NZs, in which PB serves as the substrate, has advanced rapidly.[8] Existing studies have preliminarily explored the structure‐activity relationship of PB C‐NZs. The catalytic performance is enhanced by the affinity of the dual enzyme structure and the expression of multi‐enzyme activity of cascade nanozymes.[9, 10] The interfacial electric field and chemical bond “bridge” promote electron transfer in the catalytic process.[11, 12] In addition, the presence of dual active sites (Ce^3+^/Ce^4+^ and Mn^3+^/Mn^2+^) and charge‐mediated modulation of electron transfer and circulation in the composite structure can significantly optimize the catalytic activity of the composite structure.[13, 14] These indicate that electron transfer lies at the core of redox enzyme function, and highlight the potential of structural design to enhance electron transfer and thereby improve catalytic performance, providing a substantial research foundation for the rational construction of PB C‐NZs.[15, 16] Although the reported PB C‐NZs exhibit excellent catalytic performance by increasing catalytic sites and facilitating electron transfer through optimized pathways, further improvements remain possible when structural configuration and local electronic effects are considered. Meanwhile, many pathological conditions require the cooperative action of multiple enzyme‐like activities to achieve effective therapeutic effects.[17, 18, 19] Consequently, precise regulation of catalytic behavior and expression of multi‐enzyme activities via electronic effects remains a significant challenge.[20, 21] To address this issue, a precise design strategy is necessary to fully exploit the intra‐ and inter‐nanozyme electron transfer properties fully, thereby enhancing electron transfer efficiency and regulating the redox properties of PB C‐NZs. Notably, the adjustment of interfacial electronic effects and energy band positions can effectively regulate the redox properties. Nevertheless, the underlying mechanisms by which band structural differences mediate interfacial electron transfer and promote multi‐enzyme mimetic activity remain poorly understood. Therefore, elucidating the influence of energy band position on catalytic performance and enhancing the expression of multi‐enzyme activity by adjusting redox properties can not only enable the precise design of PB C‐NZs, but also be essential for the precise and efficient application of nanozymes in diverse biomedical fields.

In our previous work, we elucidated the electron transfer catalytic mechanism and catalytic pathway of PB.[22, 23] Accordingly, the design of related PB C‐NZs should consider the electronic structure and band positions to enable precise structural tailoring for applications in various biological environments. It is well established that electron transfer is closely related to the band gap and band position of a compound.[24] Furthermore, the band positions of individual components within the composite structure significantly affect the electron transfer pathway during catalysis, thereby influencing the expression of enzyme‐like activity.[25, 26, 27, 28] In short, selecting compounds with the exact electron transfer mechanism and appropriate band positions is essential for constructing PB C‐NZs. MoS_2_ exhibits a metal valence state (Mo^6+^/Mo^4+^) similar to that of PB (Fe^3+^/Fe^2+^), and its relatively abundant redox electrons endow it with excellent electron transport properties.[29, 30] Notably, in the heterogeneous structure of MoS_2_/Vo‐Fe_2_O_3_, interfacial electron transfer from Mo to Fe significantly enhances electron conductivity.[12] Similarly, in the structure of MIL‐101 (Fe)/MoS_2_, the Mo^6+^/Mo^4+^ site can accelerate the cycle of Fe^3+^/Fe^2+^ in the catalytic process.[31] Therefore, the abundant redox electron pairs in MoS_2_ and its ability to promote electron transfer through interfacial effects make it an excellent compound for constructing PB C‐NZs.

With these considerations in mind, we have developed a novel PB C‐NZs, MoS_2_/PB, designed based on the electron transfer mechanism and transmission pathway. In this structure, MoS_2_ is employed as the substrate, and PB is grown on its surface via a surface assembly approach. In addition to facilitating intrinsic electron transfer during catalysis, the relatively high energy band position of MoS_2_ enables interfacial electron transfer toward PB, thereby enhancing the catalytic performance of the composite structure in specific reduction enzyme‐like reactions.[32] Simultaneously, the band overlap between MoS_2_ and PB effectively reduces the band gap, further enhancing electron transfer ability and improving the expression of enzyme‐like activity.[33] The prepared MoS_2_/PB thus reflects the intrinsic electron transfer behavior of PB.[14] More importantly, this composite achieves a certain degree of enhancement in reductase‐like activity through interfacial energy band differences, which, to our knowledge, is a design strategy that has not been reported before. Based on this construction mechanism, we have demonstrated the feasibility of the design concept for precisely enhancing the oxidase‐like activity of PB C‐NZs.[34] These perspectives on the design concept offer a broader framework for the rational design of PB C‐NZs with tunable electronic properties and advanced catalytic functions.

## 2 Results and Discussion

### 2.1 Preparation and Characterization of the PB Composite Nanozyme‐MoS_2_/PB

**FIGURE 1:**
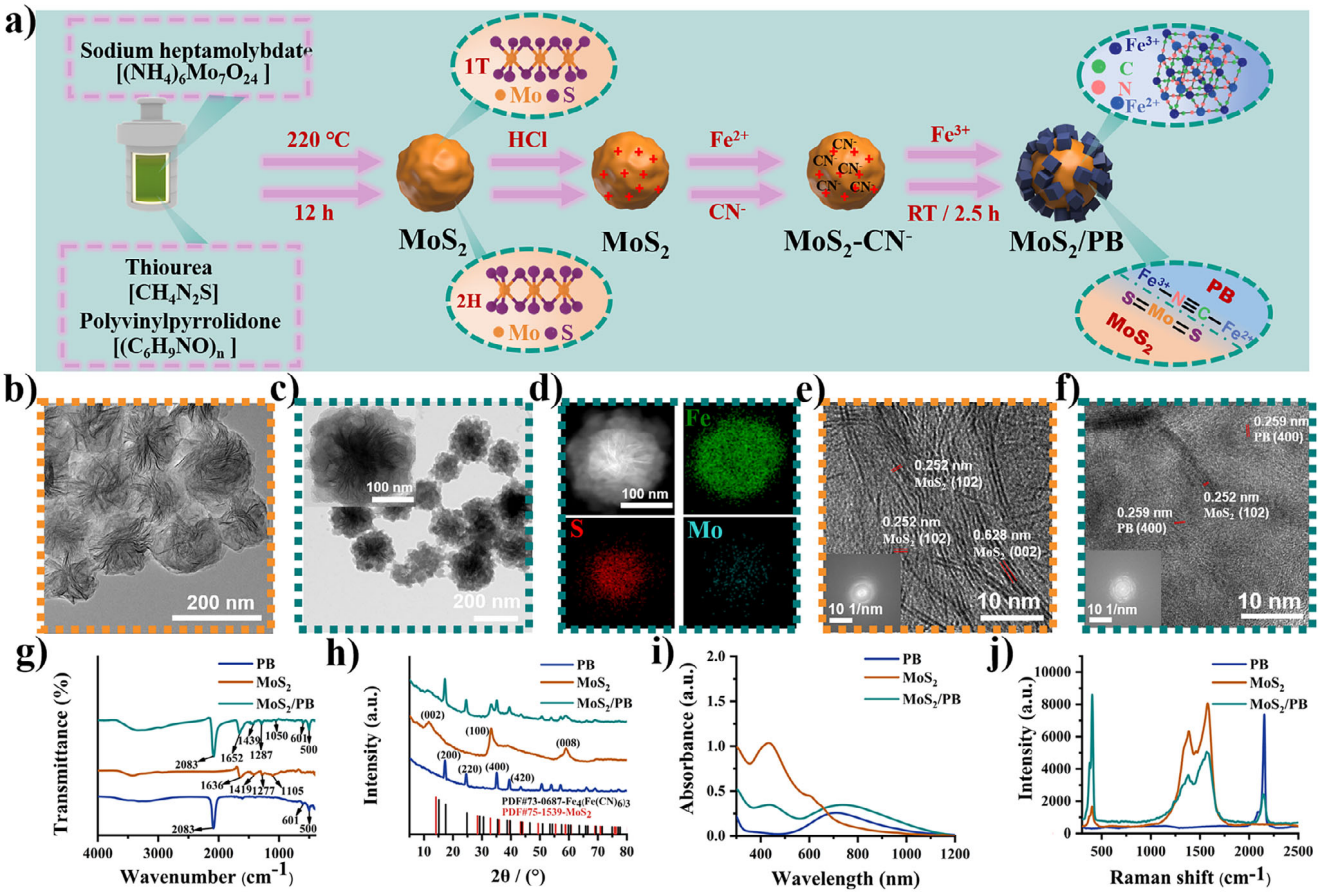
(a) Schematic presentation of the generation of MoS_2_/PB. (b) and (c) TEM images of MoS_2_ and MoS_2_/PB. (d) HAADF‐STEM‐EDS images of MoS_2_/PB. (e) and (f) AC‐HRTEM images of MoS_2_ and MoS_2_/PB. (g) FT‐IR, (h) XRD, (i) UV–vis, and (j) Raman spectrum (pattern) of PB, MoS_2_, and MoS_2_/PB.

A two‐step strategy was adopted to achieve the growth of PB on the surface of MoS_2_, which served as the substrate, through a surface assembly approach, thereby constructing the MoS_2_/PB composite nanozyme (Figure 1a). Briefly, MoS_2_ with nanoflower morphology was first synthesized as a precursor using a hydrothermal method.[35] After purification, PBNZs were grown on their surface through coprecipitation. During the growth, PB modified the partial surface electronic structure of MoS_2_. The successful preparation of the composite was initially confirmed through morphological characterization. As shown in Figure 1b and Figure S1a, both Transmission Electron Microscope (TEM) images and Scanning Electron Microscope (SEM) images reveal that the prepared MoS_2_ exhibits a spherical nanoflower morphology. After surface assembly, distinct cubic structures approximately 30 nm in size were observed on the surface of the nanoparticles (Figure S1b,c). The original MoS_2_ structure is covered on the surface to form a new nanoflower morphology (Figure 1c). The TEM image of PB obtained under the identical preparation conditions is shown in Figure S1d. To further validate the proposed surface assembly morphology of MoS_2_/PB, High‐Angle Annular Dark Field Scanning Transmission Electron Microscopy (HAADF‐STEM) was employed to observe the contrast differences. The results clearly indicate that the central region of the composite exhibits a higher contrast, which is consistent with the difference in atomic numbers between Fe and Mo (Figure S2).

Additionally, the results of the Energy Dispersive Spectroscopy STEM (EDS‐STEM) further confirmed the successful synthesis of MoS_2_/PB (Figure 1d). To investigate the microscopic structure of the nanomaterial, the Aberration Corrected High Resolution TEM (AC‐HRTEM) was selected. As shown in Figure 1e,f, the AC‐HRTEM image of MoS_2_ displays lattice spacings attributed to MoS_2_. In contrast, the image of the composite material MoS_2_/PB reveals the lattice spacings corresponding to both MoS_2_ and PB. Specifically, the lattice spacings of 0.252 nm and 0.628 nm correspond to the (002) and (102) planes of MoS_2_, respectively, while the lattice spacing of 0.259 nm corresponds to the (400) plane of PB.[36, 37] Furthermore, the Fast Fourier Transform of AC‐HRTEM images demonstrates corresponding changes in crystal information (Figure S3). Elemental analysis provided further confirmation of the successful formation of the composite structure, as characteristic signals corresponding to Fe, Mo, and S were clearly detected (Figure S4). In summary, we successfully prepared the MoS_2_/PB nanocomposite, and the optical image of its aqueous solution is presented in Figure S5.

### 2.2 Structural Components and Crystal Structure

To further verify the successful synthesis of MoS_2_/PB, Fourier Transform Infrared (FTIR) spectroscopy was performed on the composite material as well as on each component. The FTIR spectra of MoS_2_, PB, and MoS_2_/PB exhibit significant differences (Figure 1g). Before assembly, MoS_2_ shows characteristic absorption bands at 1105, 1277, 1419, and 1636 cm^−1^. The bands at 1105, 1277, and 1636 cm^−1^ are assigned to the stretching vibration of the C─O group, O─C─O group, and C═O group of polyvinylpyrrolidone (PVP), respectively. Additionally, the band at 1419 cm^−1^ corresponds to the stretching vibration of the S−Mo−S bond.[38] After surface assembly of PB, the out‐of‐plane and in‐plane bending vibrations of the Fe─C bond are observed at 500 and 601 cm^−1^. Moreover, a strong C≡N stretching vibration of Fe─C≡N─Fe is observed at 2083 cm^−1^, which is characteristic of the PB structure. The peak representing the S─Mo─S bond at 1419 cm^−1^ exhibits a red shift, which can mainly be attributed to the introduction of CN^−^ during the formation of PB.[39] Moreover, the change in Zeta potential before and after assembly further confirms the growth of PB on the MoS_2_ surface (Figure S6). To further investigate the crystal composition of MoS_2_/PB, X‐ray diffraction (XRD) analysis was performed. As shown in Figure 1h, the characteristic diffraction peaks of MoS_2_/PB are located at 11.73°, 33.20°, and 59.93°, corresponding to the (002), (100), and (008) lattice planes of MoS_2_.[40] Additionally, the characteristic diffraction peaks at 17.19°, 24.65°, 35.27°, and 39.55° are observed, which are attributed to the (200), (220), (400), and (420) lattice planes of PB.[41] The intensity of the characteristic peak corresponding to MoS_2_ is reduced, likely due to the growth of PB on its surface, which partially covers the crystal information of MoS_2_. To determine the crystalline phase of MoS_2_, Ultraviolet‐Visible (UV–vis) and Raman spectroscopy were performed. The UV–vis spectrum shows three characteristic peaks at 431, 609, and 668 nm. Given that the 1T‐MoS_2_ lacks UV–vis absorption features, MoS_2_ is preliminarily confirmed to have a 2H crystalline phase (Figure S7).[42] The MoS_2_/PB curve shows a characteristic peak of PB at 700 nm after surface assembly (Figure 1i). Additionally, the Raman spectrum of MoS_2_/PB reveals three characteristic peaks, one of which, 2153.4 cm^−1^ corresponds to C≡N (Figure 1j and Figure S8a). A broader characteristic peak in the range of 1200–1800 cm^−1^ is attributed to PVP outside MoS_2_ (Figure S8b,c). Importantly, two characteristic Raman peaks at 378.7 cm^−1^ (E^1^_2g_) and 403.9 cm^−1^ (A_1g_) are observed in both MoS_2_ and MoS_2_/PB, further confirming the existence of the 2H‐MoS_2_ phase (Figure S8d).[42] These results confirm that MoS_2_/PB with high phase purity and high crystallinity has been successfully prepared.

Although the information regarding the preparation of MoS_2_/PB is clear, its assembly process requires further investigation. The preparation strategy of MoS_2_/PB leverages the fact that the surface electronic structure of MoS_2_ changes under acidic conditions, influencing the charge dynamics.[43] While the MoS_2_ is exposed to acidic conditions, a positive charge is generated on its surface. This results in the electrostatic adsorption of CN^−^, which subsequently reacts with the Fe^2+^ and Fe^3+^, facilitating the PB assembly on the MoS_2_ surface. To validate this hypothesis, MoS_2_ was analyzed under acidic conditions. After adding K_4_[Fe(CN)_6_] and removing the added molecules, the results show that as acidity increases, the Zeta potential of MoS_2_ shifts in the positive direction. Notably, this potential can be restored to its original value when the solution returns to neutral (Figure S9a,b). After the addition of K_4_[Fe(CN)_6_], the potential change trend remains consistent, and the 2H crystal phase of MoS_2_ is preserved (Figure S9c,d). Furthermore, Raman spectroscopy and TEM images further confirm the crystal stability of MoS_2_ under these preparation conditions (Figures S10,S11). These findings provide comprehensive evidence supporting the composition, crystal structure, and formation process of MoS_2_/PB.

### 2.3 Examinations of Microstructure and Electronic Structure

**FIGURE 2:**
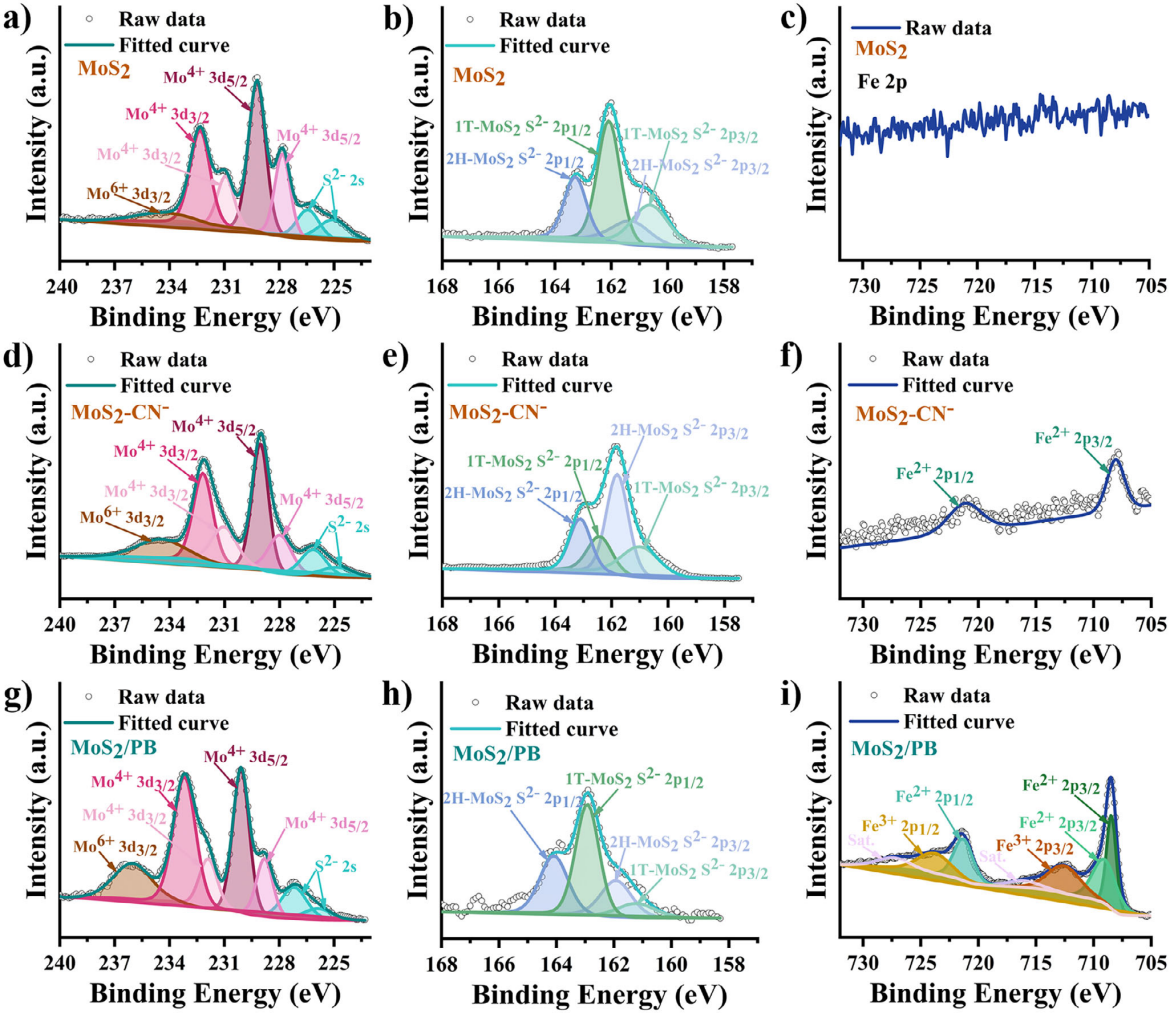
High‐resolution XPS spectra of MoS_2_, MoS_2_‐CN^−^, and MoS_2_/PB. (a), (d), and (g), Mo 3d. (b), (e), and (h), S 2p. (c), (f), and (i), Fe 2p.

The enzyme‐like catalytic mechanism of PB is predominantly governed by the electron transfer processes, so the electronic structure and valence state characteristics of PB C‐NZs are crucial for elucidating their catalytic behavior. To further probe the structural modifications induced by PB surface assembly and to examine the surface chemical composition of the MoS_2_/PB composite, X‐ray photoelectron spectroscopy (XPS) was employed. Additionally, the result can also be used to further analyze whether there is a 1T crystal phase in the MoS_2_ structure. The XPS spectrum of MoS_2_, MoS_2_‐CN^−^, and MoS_2_/PB, shown in Figure 2, provides insights into the local electronic structure and valence state information. Six main peaks are observed at binding energies of 163, 231, 283, 399, 531, and 708–728 eV, corresponding to S 2p, Mo 3d, C 1s, N 1s, O 1s, and Fe 2p, respectively (Figure S12a).[44, 45]

The XPS spectrum of the Mo element in the MoS_2_ structure reveals seven distinct spectral bands after curve fitting, among which the peaks at 225 and 227 eV with lower binding energy are attributed to S^2−^ 2s (Figure 2a).[45] The valence states of the Mo element in the MoS_2_ spectrum include Mo^4+^ 3d_5/2_, Mo^4+^ 3d_3/2_, and Mo^6+^ 3d_3/2_, reflecting the sulfidation state of Mo species in the synthesized MoS_2_ structure. Specifically, the peaks centered at 227.8 and 229.2 eV are attributed to the Mo^4+^ 3d_5/2_, while the peaks centered at 230.9 and 232.3 eV correspond to Mo^4+^ 3d_3/2_. The peak centered at 234.4 eV is assigned to the Mo^6+^ 3d_3/2_, consistent with previously reported MoS_2_ spectra.[42, 46] Therefore, the Mo in the structure exists in two sulfidation states, Mo^4+^ and Mo^6+^, whose coexistence will form relatively independent electron cycling states contributing to enzyme‐mimicking activity.[47] Furthermore, the XPS spectrum of the S element in MoS_2_ reveals four distinct spectral bands, corresponding to 1T‐MoS_2_ S^2−^ 2p_3/2_, 1T‐MoS_2_ S^2−^ 2p_1/2_, 2H‐MoS_2_ S^2−^ 2p_3/2_, and 2H‐MoS_2_ S^2−^ 2p_1/2_, which reflect the valence states of sulfur species and the crystal phase information of MoS_2_ (Figure 2b). So, the Mo 3d and S 2s spectra indicate the coexistence of both the 2H phase and a 1T phase of MoS_2_.[48] The peaks centered at 160.7 and 162.1 eV are attributed to 1T‐MoS_2_ S^2−^ 2p_3/2_ and 1T‐MoS_2_ S^2−^ 2p_1/2_, respectively, while the peaks centered at 161.3 and 163.3 eV correspond to 2H‐MoS_2_ S^2−^ 2p_3/2_ and 2H‐MoS_2_ S^2−^ 2p_1/2_, respectively. In contrast, the XPS spectrum does not display any identifiable peaks of Fe, confirming the absence of Fe‐related spectral information in the MoS_2_ structure (Figure 2c).

To further verify the stable adsorption of CN^−^ on the MoS_2_ surface, XPS analysis of MoS_2_‐CN^−^ was performed, and the overall spectrum is presented in Figure S12b. Notably, in the MoS_2_‐CN^−^, the partial binding energies of both Mo and S orbitals are shifted compared to MoS_2_. (Figure 2d,e). The specific values ​​are shown in Tables S1,S2. This should be caused by the surface adsorption of CN^−^ and Fe^2+^. Among the N 1s spectra, four bands are revealed, with four peaks centered at 394.8, 397.2, 399.4, and 400.1 eV, corresponding to the bonding states of C─N, C≡N, Mo─N, and PVP─N.[44] The presence of a C≡N peak at 397.2 eV provides strong evidence that CN^−^ can be adsorbed on the MoS_2_ surface, facilitating subsequent assembly processes (Figure S13a,b). Additionally, the N spectrum of MoS_2_/PB reveals the characteristic peak of C≡N at 397.8 eV, which is absent in the spectrum of MoS_2_ (Figure S13c). This finding aligns with the previous analysis of the assembly process, further confirming the incorporation of PB into the composite structure. Simultaneously, the overall spectrum does not show a prominent peak for the Fe 2p orbital. However, upon further analysis, peaks are observed at 708.1 and 721.2 eV (Figure 2f), corresponding to Fe^2+^ 2p_3/2_ and Fe^2+^ 2p_1/2_, respectively. This phenomenon is likely due to the interaction of CN^−^ with the MoS_2_ surface or the adsorption of free Fe^2+^ onto unsaturated sulfur sites.

The electronic structure of the MoS_2_/PB surface plays a crucial role in the catalytic process. The XPS spectrum of the Mo element reveals seven distinct spectral bands after curve fitting, with the peak at the lower binding energy attributed to S^2−^ 2s. Notably, the valence states of the Mo in the MoS_2_/PB structure remain consistent, exhibiting two valence states, Mo^4+^ 3d_5/2_, Mo^4+^ 3d_3/2_, and Mo^6+^ 3d_3/2_ (Figure 2g). Specifically, the peaks centered at 228.8 and 230.1 eV correspond to Mo^4+^ 3d_5/2_, while the peaks at 231.9 and 233.2 eV correspond to Mo^4+^ 3d_3/2_. The peak centered at 236.0 eV is attributed to the Mo^6+^ 3d_3/2_. Similarly, four distinct spectral bands are observed in the XPS spectrum of the S element of MoS_2_/PB (Figure 2h). The peaks at 161.2 and 162.9 eV are assigned to 1T‐MoS_2_ S^2−^ 2p_3/2_ and 1T‐MoS_2_ S^2−^ 2p_1/2_, respectively, while the peaks centered at 161.9 and 164.1 eV are attributed to the 2H‐MoS_2_ S^2−^ 2p_3/2_ and 2H‐MoS_2_ S^2−^ 2p_1/2_, respectively.[45] It is noteworthy that, in the MoS_2_/PB structure, the binding energies of both Mo and S orbitals exhibit a noticeable shift toward higher binding energy compared to those in MoS_2_ (Table S1,S2). The results indicate the presence of electron‐withdrawing groups in the surrounding environment, which can be attributed to the assembly of PB on the MoS_2_ surface.[49]

As expected, the Fe spectrum of MoS_2_/PB exhibits significant features, with seven distinct spectral bands identified after curve fitting (Figure 2i). Among these, the peaks at 716.9 and 727.6 eV are satellite peaks. The remaining five peaks correspond to the characteristic Fe 2p signals, reflecting the valence states of the iron element in PB within the MoS_2_/PB composite. Specifically, the three peaks centered at 708.5, 709.1, and 721.3 eV are attributed to Fe^2+^ 2p_3/2_, Fe^2+^ 2p_3/2_, and Fe^2+^ 2p_1/2_, respectively. Meanwhile, the two peaks centered at 712.5 and 723.8 eV are attributed to the Fe^3+^ 2p_3/2_ and Fe^3+^ 2p_1/2_, respectively. Notably, the proportion of Fe^2+^ in the surface structure is relatively high, consistent with most current reports [23]. Furthermore, the valence distribution of the Fe in PB synthesized using the same method is also in agreement (Figure S14). Interestingly, the binding energy positions of the Fe element's different valence states exhibit a shift toward lower binding energies, suggesting the presence of electron‐donating groups in the surrounding environment (Table S3).[49] This should be caused by the internal MoS_2_. The offset in binding energy of the two further illustrates the success of the assembly.

What is particularly noteworthy is the significant increase in the content of Mo^6+^ within the MoS_2_ structure after treatment or assembly. Specifically, the proportion of Mo^6+^ in MoS_2_ is 6% of the Mo content, whereas in MoS_2_/PB, it rises to 16% (Table S4). Analysis of MoS_2_ at different stages suggests that this phenomenon is likely due to partial oxidation of the MoS_2_ surface under acidic conditions, resulting in an increased Mo^6+^ content (Figure S15).[50] Importantly, a higher Mo^6^^+^ content is associated with the increased availability of electron transfer–active sites, which may facilitate charge transport and thereby contribute to the enhanced activity observed in certain enzyme‐mimicking reactions.

### 2.4 Electron Transport and Catalytic Mechanisms

Electron transfer capability is a critical determinant of the catalytic performance of PB‐based nanozymes. Moreover, elucidation of the electron transfer pathways is essential for gaining insight into their enzyme‐mimicking catalytic behavior. Therefore, UV–visible Diffuse Reflectance Spectroscopy (DRS) was employed to calculate the band gap (Eg) value (Figure S16). Subsequently, the valence band (VB) and conduction band (CB) positions were determined using UV Photoelectron Spectroscopy (UPS), enabling a detailed analysis of the difficulty and pathways of the electron transfer.

**FIGURE 3:**
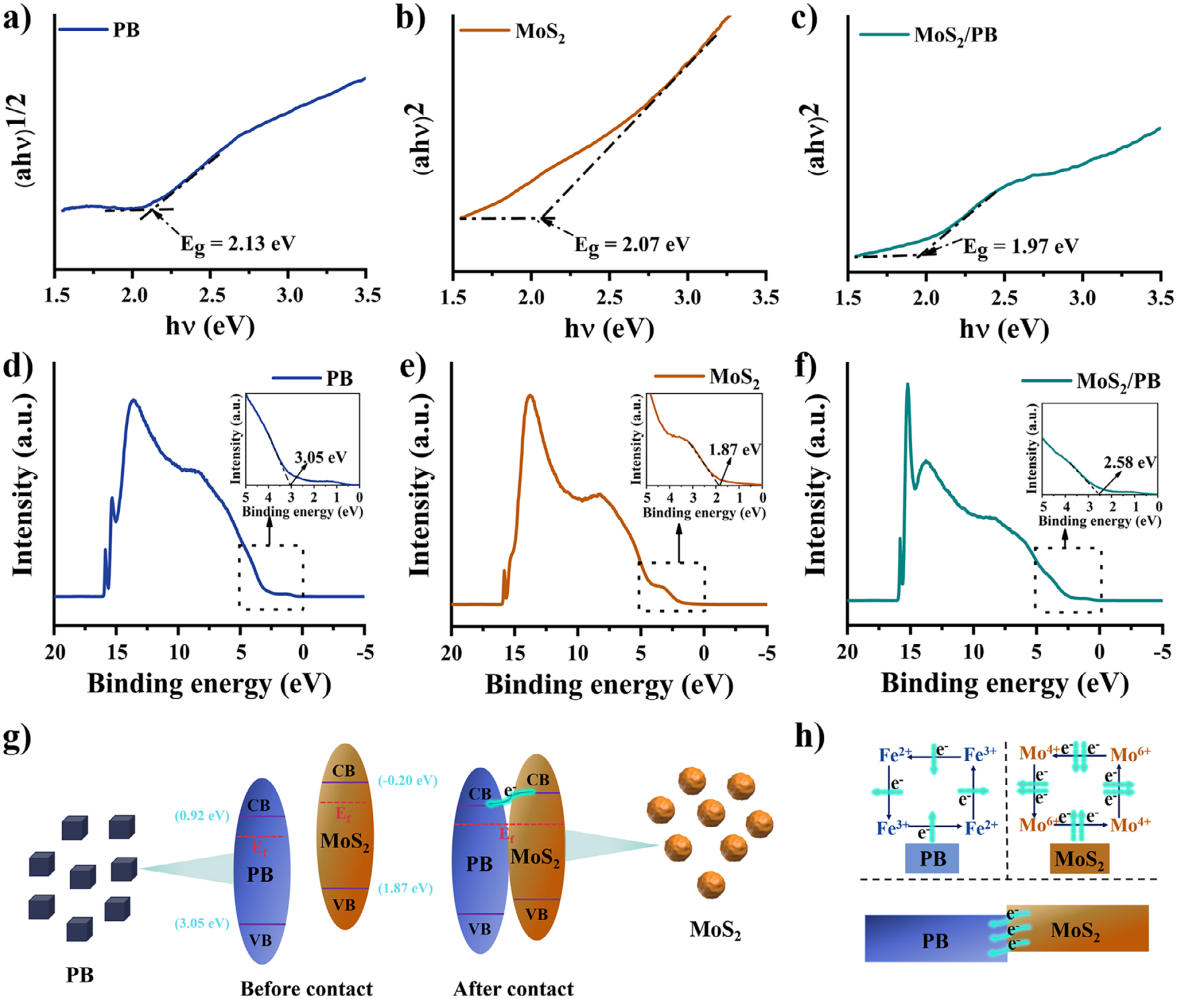
Bandgap of PB (a), MoS_2_ (b), and MoS_2_/PB (c). UPS spectra of PB (d), MoS_2_ (e), and MoS_2_/PB (f). (g) Schematic diagram of electron transfer. (h) Schematic diagram of the electron transport pathway.

The Eg values of PB, MoS_2,_ and MoS_2_/PB were estimated to be 2.13, 2.07, and 1.97 eV, respectively, demonstrating that the combination of PB and MoS_2_ slightly reduces Eg (Figure 3a–c). This reduction in Eg facilitates easier electron movement within the composite structure.[51] Furthermore, the UPS results reveal that the VB binding energies of PB, MoS_2_, and MoS_2_/PB are 3.05, 1.87, and 2.58 eV, respectively (Figure 3d–f), allowing for the determination of the corresponding energy band positions (Figure S17). Specifically, the valence band potential (E_VB_) and conduction band potential (E_CB_) of PB are approximately 3.05 and 0.92 eV, respectively, while those of MoS_2_ are approximately 1.87 and −0.20 eV, respectively. For MoS_2_/PB, the E_VB_ and E_CB_ are approximately 2.58 and 0.61 eV, respectively. Notably, the E_CB_ of MoS_2_/PB shifts in the positive direction compared to PB, a change that is anticipated to enhance the catalytic effect of specific reduction enzyme‐like reactions. In summary, the composite structure of MoS_2_/PB not only promotes electron transfer to enhance enzyme‐like activity but also has the potential to improve the reductive enzyme‐like activity of PB.[52] As shown in Figure 3g, the positions of VB and CB indicate that PB and MoS_2_ can promote electron transfer because of their overlapping energy band potentials. Notably, the CB potential of MoS_2_ is more negative, enabling electrons from MoS_2_ to easily migrate to the surface of PB, resulting in interfacial electron transfer.[53] More importantly, since the mechanism of the related enzymatic reaction is redox‐based, MoS_2_/PB can acquire electrons from the reaction, thereby supporting a catalytic cycle. Consequently, the electron transfer pathway of MoS_2_/PB is illustrated in Figure 3h. Within the composite structure, two relatively independent intra‐electron cycles are established—one associated with PB and the other with MoS_2_.[54] Additionally, some electrons migrate from MoS_2_ to the PB surface, generating interfacial electron transfer, further enhancing the catalytic performance.

### 2.5 Density Functional Theory (DFT) on the Interfacial Electron Transfer of MoS_2_/PB

**FIGURE 4:**
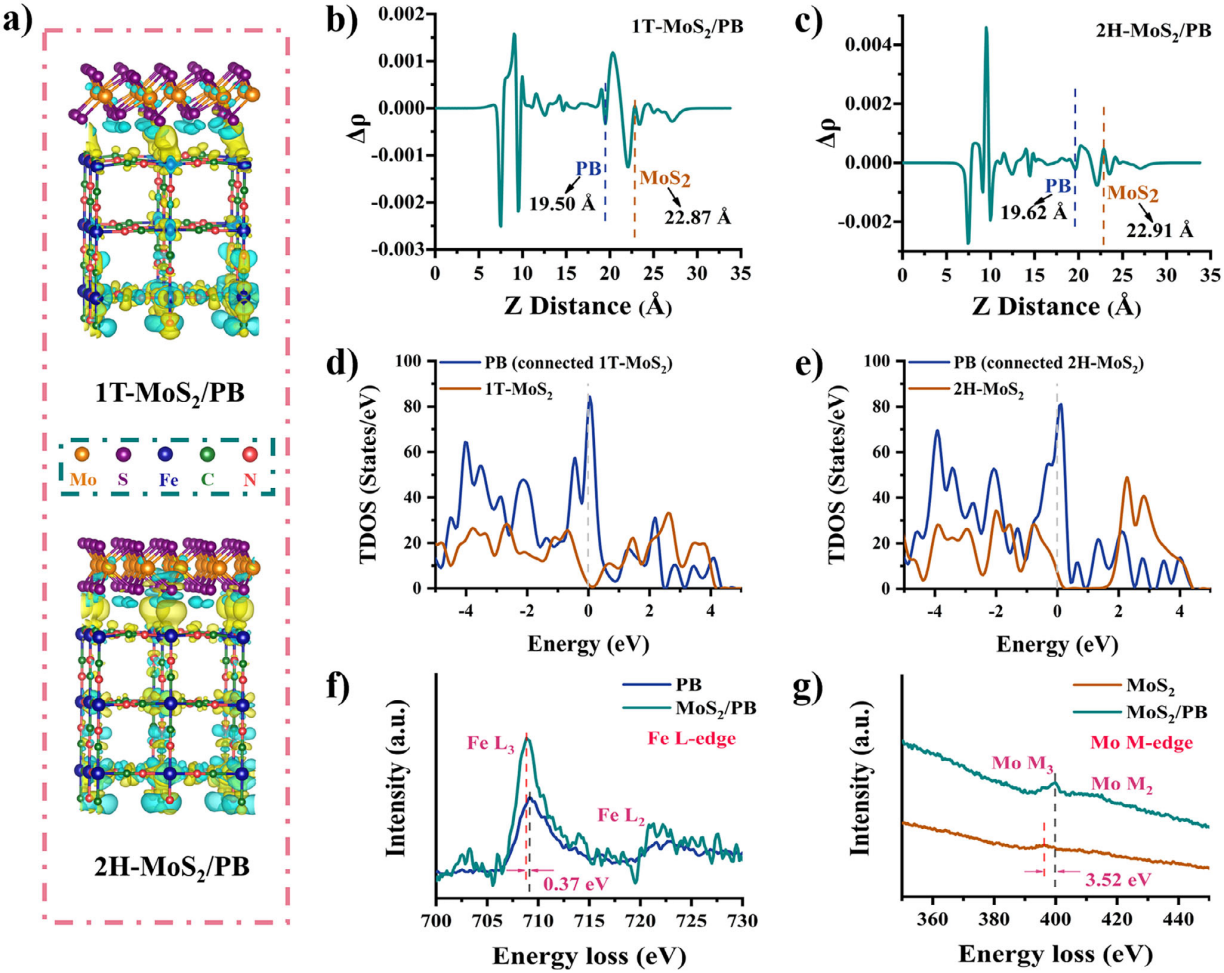
(a) Differential charge density (DCD) structure diagram of 1T‐MoS_2_/PB and 2H‐MoS_2_/PB. The yellow and cyan areas represent the gain and loss of electrons, respectively. (b) and (c) Differential charge Z‐direction plane average charge density of 1T‐MoS_2_/PB and 2H‐MoS_2_/PB. (d) and (e) Total density of states (TDOS) of 1T‐MoS_2_/PB and 2H‐MoS_2_/PB. (f) EELS spectra of the Fe L‐edge. (g) EELS spectra of the Mo M‐edge.

For composite nanozymes that operate via electron transfer mechanisms, interfacial electronic effects play a crucial role in governing the expression of enzyme‐like activities. Given that MoS_2_ is confirmed to consist of both 1T and 2H crystalline phases, the coexistence of these phases is expected to modulate interfacial charge transfer behavior, thereby influencing the catalytic performance of the composite nanozyme. Therefore, in order to verify the interfacial electron transfer effect between MoS_2_ and PB, DFT calculations were performed on the two interfacial electronic environments of 1T‐MoS_2_/PB and 2H‐MoS_2_/PB, and the optimized crystal structure is shown in Figure S18. The results of differential charge density calculations show that obvious charge transfer can be observed at the interface for both 1T‐MoS_2_ and 2H‐MoS_2_ crystals, indicating that the MoS_2_/PB interface can effectively transfer electrons from MoS_2_ to PB (Figure 4a).[55, 56] The energy band arrangement provides another theoretical support for this transfer. It is worth noting that after the interfacial electron transfer occurs, the valence state of Fe ions near the interface also changes. According to our previous research, it can be concluded that this interfacial effect is consistent with the electron transfer mechanism of PBNZs; that is, this interfacial effect can be used to increase the electron transfer path. This phenomenon is expected to improve the expression of enzyme‐like activity. Simultaneously, this valence state conversion of the adjacent interface may also bring about a longer‐lasting and stable interfacial electron transfer efficiency. It can be seen from the charge density diagram mapped to the z‐axis that, compared with 2H‐MoS_2_, 1T‐MoS_2_ shows a more favorable electron transfer from MoS_2_ to PB (Figure 4b,c). The Bader charge meter shows that 1T‐MoS_2_ also transfers 0.24 e to PB, while 2H‐MoS_2_ transfers 0.12 e to PB. This difference can also be verified in the simulation results of the work function (Figure S19).[34] Meanwhile, theoretical analysis of the density of states further confirms the above results. Simply put, in both the 1T‐MoS_2_/PB and 2H‐MoS_2_/PB structures, PB occupies the portion of the unoccupied states closest to the Fermi level, making it easier for PB to gain electrons to fill these unoccupied states. For MoS_2_, the occupied states in 2H‐MoS_2_ are shifted to the left compared to 1T‐MoS_2_, so electrons from MOS_2_ in the 1T system are more easily transferred to the PB structure (Figure 4d,e).[57] Further calculations of the PDOS for both structures show that the unoccupied states of PB are mainly contributed by Fe, while the occupied states of MoS_2_ are mainly composed of Mo. Therefore, during electron migration, the electrons mainly migrate from the occupied states of Mo to the unoccupied states of Fe in PB (Figure S20). This difference between different crystal phases will also provide new ideas for the design of subsequent composite nanozymes. In addition, electron energy loss spectroscopy (EELS) reveals a significant red shift in the Fe L_3_ edge and a corresponding blue shift in the Mo M_3_ edge in MoS_2_/PB (Figure 4f,g, and Figure S21). These spectral shifts provide strong evidence for the interfacial electron transfer from Fe to Mo.[58, 59, 60] Furthermore, surface photovoltage (SPV) and transient photovoltage (TPV) measurements demonstrate that the interfacial architecture of MoS_2_/PB generates a driving force for electron migration (Figure S22).[61, 62, 63] In short, whether it is 1T‐MoS_2_ or 2H‐MoS_2_, its electron migration to PB will drive a more efficient reduction enzyme‐like properties.[64] This is also in line with the design concept of improving the catalytic performance of reducing enzymes and enriching the expression of multi‐enzyme activities through intra‐ and inter‐nanozyme electron transfer (Figure S23).

### 2.6 Simulate Multi‐Enzyme Activity

After determining the surface electronic structure and energy band information of MoS_2_/PB, its enzymatic activity was evaluated as a novel PB C‐NZs. PB C‐NZs are primarily known for their excellent oxidation/reduction properties, which enable them to regulate biological signaling molecules within the microenvironment of organisms for therapeutic purposes.[65] In this study, a simulation‐based method was employed to assess the activities of six related enzymes: catalase (CAT), peroxidase (POD), superoxide dismutase (SOD), glutathione peroxidase (GSH‐Px), S‐nitrosoglutathione reductase (GSNOR), and nitrite reductase (NiRs). Notably, the enzymatic activities of GSNOR and NiRs have not been previously reported for PB C‐NZs. The detection principles and methods were consistent with those described previously.[66, 67, 68]

**FIGURE 5:**
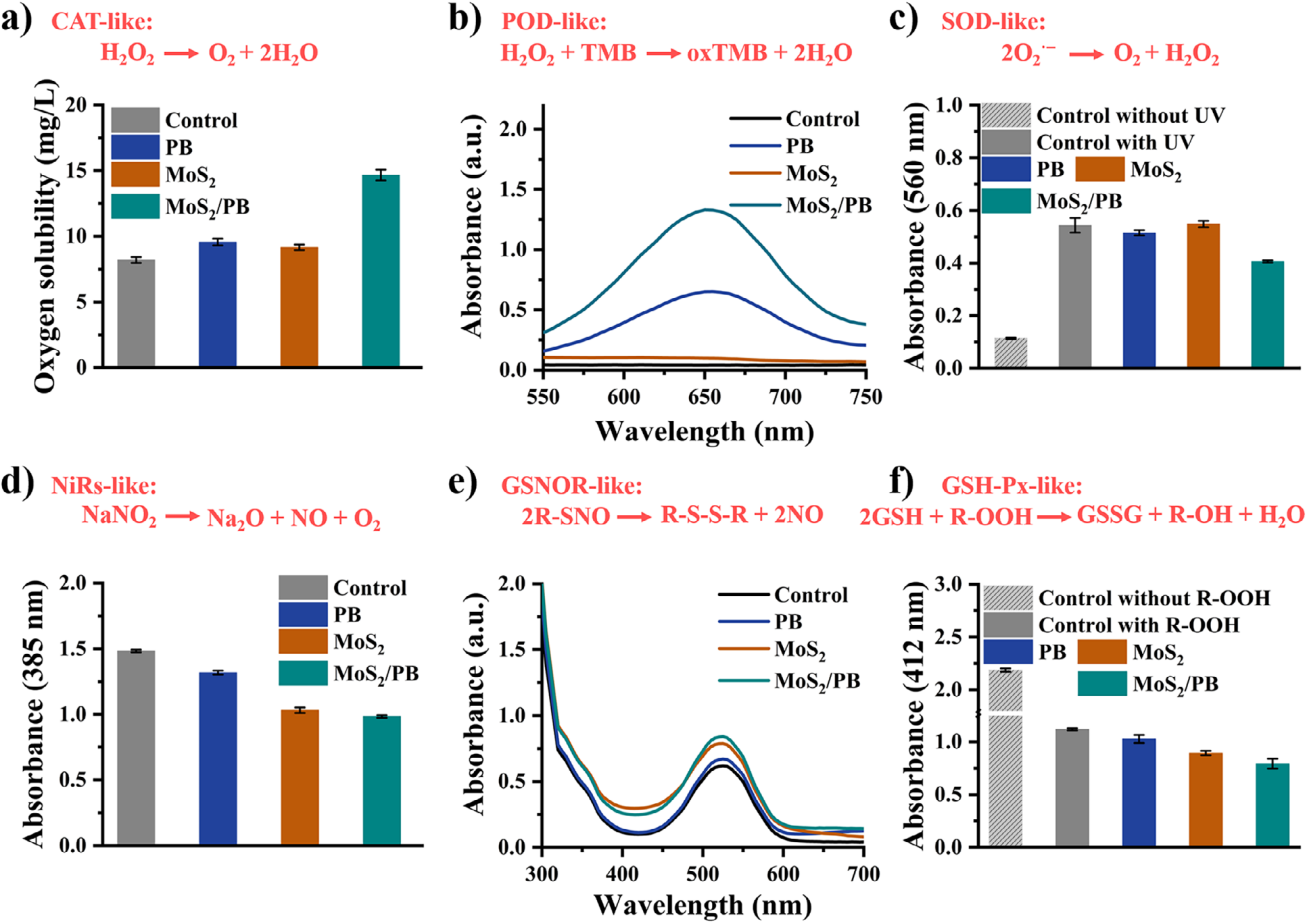
Multi‐enzyme‐like activity results of PB, MoS_2,_ and MoS_2_/PB. (a) CAT, *n* = 3. (b) POD. (c) SOD, *n* = 3. (d) NiRs, *n* = 3. (e) GSNOR. (f) GSH‐Px, *n* = 3.

To more explicitly demonstrate that the enhanced enzymatic activity of MoS_2_/PB is due to electron transfer, 2‐hydroxybenzoic acid was chosen to investigate whether it is affected by the Fenton reaction. The results are shown in Figure S24 and reveal that neither MoS_2_/PB nor MoS_2_ and PB alone exhibit any Fenton reaction, and free Fe^2+^ does not interfere with the reaction. Therefore, the enhanced electron transfer efficiency, resulting from the unique electron transfer mechanism in the structure, will be the fundamental reason for the excellent enzyme‐like activity of MoS_2_/PB. To evaluate the enzymatic activity of MoS_2_/PB, the previously reported method was first employed to assess CAT activity. As shown in Figure 5a, MoS_2_/PB demonstrated superior CAT‐like activity compared to both PB alone and single‐component MoS_2_. The catalysis performance of MoS_2_/PB at varying concentrations toward hydrogen peroxide revealed that MoS_2_/PB maintained its excellent CAT‐like activity (Figure S25). Next, the POD‐like activity of MoS_2_/PB was examined. The results showed that MoS_2_/PB also exhibited highly efficient enzyme‐like activity (Figure 5b and Figure S26). The enhanced POD‐like activity of MoS_2_/PB was attributed primarily to the improved electron transfer ability of PB and the composite structure. Furthermore, the SOD activity, which is crucial for scavenging ROS and providing anti‐inflammatory effects, was also tested. As anticipated, MoS_2_/PB displayed significant SOD‐like activity (Figure 5c). Even with increasing concentration or prolonged reaction times, the composite maintained stable SOD‐like activity (Figure S27). In contrast, single‐component MoS_2_ exhibited negligible SOD‐like activity. This observation underscores the role of the MoS_2_/PB composite structure in enhancing enzyme‐mimicking activity through intra‐ and inter‐nanozyme electron transfer. Furthermore, the synergistic effect of inter‐nanozyme can be further observed through POD‐like kinetics (Figure S28).

The three enzyme‐mimicking activities are primarily attributed to PB nanozymes, whereas MoS_2_ in the composite mainly serves to enhance electron transfer efficiency and introduce synergistic effects. Therefore, to comprehensively elucidate the enzyme‐like catalytic properties of MoS_2_/PB composite, special attention should be paid to the catalytic activities that are weak or absent in PB nanozymes. Through extensive experimentation, we identified that the MoS_2_/PB composite exhibits remarkable catalytic activities analogous to glutathione peroxidase (GSH‐Px), S‐nitrosoglutathione reductase (GSNOR), and nitrite reductase (NiRs). Notably, these enzyme‐like activities are significantly enhanced in the MoS_2_/PB composite compared to the negligible activity observed in PB nanozymes.

The NiRs activity can be evaluated by detecting the nitrite concentrations. As shown in Figure S29a,b, the selected detection reagent does not interfere with the experimental results. The catalytic activity is quantified by measuring absorbance at 385 nm, where the lower absorbance corresponds to higher catalytic efficiency. Figure 5d demonstrates that while PB exhibits the ability to promote nitrite decomposition, MoS_2_ shows a pronounced catalytic effect. Notably, the MoS_2_/PB exhibits excellent NiRs‐like activity, which increases with increasing concentration (Figure S29c).

Based on the previous valence state and energy band analyses, we hypothesize that the MoS_2_/PB composite structure will be more beneficial to the reduction enzyme‐like reaction. To verify this hypothesis, we selected two reductive enzyme‐like, GSNOR and GSH‐Px. The results of GSNOR are shown in Figure 5e. The principle of this assay is based on detecting the amount of NO released, where a higher absorbance at a specific wavelength indicates greater NO release. The results demonstrate that, compared to PB or MoS_2_, MoS_2_/PB exhibits the most vigorous GSNOR‐like activity. Similarly, its catalytic ability increases with concentration. Furthermore, MoS_2_/PB retains excellent enzymatic activity over a specific period (Figure S30). The GSH‐Px activity is determined by measuring the content of GSH, with a lower absorbance indicating lower content. The results show that MoS_2_/PB demonstrates superior GSH‐Px‐like catalytic activity compared to both PB and MoS_2_ (Figure 5f and Figure S31). The GSH‐Px‐like activity of PB alone is negligible.

The difference lies in the fact that the catalytic reactions of GSNOR and GSH‐Px are mainly rely on their reduction enzyme‐like properties, distinct from the redox enzyme‐like discussed earlier. MoS_2_ exhibits vigorous catalytic activity, likely due to the presence of Mo^4+^ in its structure, which can be converted into Mo^6+^, thereby providing more electrons. The enhanced catalytic effect of MoS_2_/PB can be attributed to the improvement in electron transfer efficiency due to multiple electron transfer pathways and the enhancement of electron transfer from MoS_2_ to PB at the interface, as well as the inter‐nanozyme electron transfer, which further improves the reduction enzyme‐like performance. These results highlight the structural advantages of the rationally designed MoS_2_/PB composite. Collectively, the enhanced catalytic performance and the multiple enzyme‐like activities confirm that our preliminary design strategy for PB‐based composite nanozymes is reasonable and effective. Notably, the multi‐enzyme‐like activities of MoS_2_/PB are primarily reflected in its ability to regulate the homeostasis of nitric oxide (NO) and glutathione (GSH). Accumulating evidence has demonstrated that these two signaling molecules play crucial roles in mediating cellular signaling pathways and maintaining redox homeostasis, thereby contributing to therapeutic outcomes in a variety of disease models.[69, 70, 71] These observations strongly highlight the considerable biomedical potential of PB‐based composite nanozymes (PB C‐NZs) constructed under the proposed design strategy.

## 3 Conclusion

In summary, we have rationally designed and constructed a novel PB C‐NZs, MoS_2_/PB, via surface assembly, guided by electron‐transfer mechanisms. The formation process was systematically investigated, and comprehensive structural and compositional characterizations were conducted. Band gap measurements and energy band position analyses confirmed that the MoS_2_/PB structure facilitates efficient electron transfer. DFT calculations further supported the electron transfer at the MoS_2_/PB interface. Notably, 1T‐phase MoS_2_ was identified as more favorable for interfacial electron transport, offering valuable insights for the future design of composite nanozymes. Interfacial electron transfer from MoS_2_ to PB significantly enhances reductive enzyme‐like catalytic activity, consistent with the observed structural features. The MoS_2_/PB composite exhibits markedly improved enzyme‐mimicking performance, particularly in reductive enzyme‐like activities such as GSNOR and GSH‐Px. Importantly, this enhancement is achieved without altering the intrinsic catalytic mechanism, owing to synergistic intra‐ and inter‐nanozyme electron transfer. In conclusion, the MoS_2_/PB integrates multiple catalytic activities, demonstrating strong potential for biomedical applications. Furthermore, the design strategy and mechanistic insights presented in this work provide a novel approach for developing high‐performance composite nanozymes.

## 4 Experimental Section

### 4.1 Preparation of MoS_2_ Nanoflower

The MoS_2_ nanoflowers were prepared by the hydrothermal method. Specifically, the raw materials were ammonium heptamolybdate as the molybdenum source, thiourea as the sulfur source, and polyvinyl pyrrolidone as the dispersant. The process was to add 60 mg of ammonium heptamolybdate, 76 mg of thiourea, and 600 mg of polyvinyl pyrrolidone to 17 mL of deionized water under constant stirring and continue stirring for 30 min after being fully dissolved. Then, it was transferred to a hydrothermal reactor of appropriate volume, heated at 220 °C for 12 h, and then slowly cooled. The resulting mixture was repeatedly centrifuged with pure water and washed several times to finally obtain MoS_2_ nanoflowers. The above solution was freeze‐dried to obtain a powder sample, which was then dissolved using ultrapure water as a solvent, and the concentration was calibrated with mass fraction.

### 4.2 Preparation of PB

The PB were prepared using a co‐precipitation method with a dual iron source strategy. Specifically, 55 mL of ultrapure water was added to the reaction vessel, and the pH was adjusted to 3 using HCl (6M) as the base solution. Subsequently, 10 mL each of two pre‐prepared solutions, K_4_[Fe(CN)_6_] and citric acid/FeCl_3_ solution, were added simultaneously. After the addition was complete, the mixture was stirred at room temperature, and the reaction was terminated after 2 h. Then, the reactants were centrifuged and washed several times to remove unreacted molecules, finally yielding PBNZs.

### 4.3 Preparation of MoS_2_/PB

The co‐precipitation method was selected to prepare PB and assemble it on the MoS_2_ surface. Specifically, the above‐mentioned fixed concentration MoS_2_ solution (20 mL) was dispersed in ultrapure water (35 mL). After being fully mixed, a certain amount of HCl was added to adjust the pH to 3. After continuous stirring for 30 min, 10 mL of the previously prepared K_4_[Fe(CN)_6_], citric acid solution, and FeCl_3_ solution were added in sequence. After being added in sequence, the mixture was stirred at room temperature, and the reaction was terminated after 2 h. Then, the reactants were centrifuged and washed several times to remove unreacted molecules, and MoS_2_/PB was finally obtained.

### 4.4 Statistical Analysis

Quantitative date were presented as the mean ± standard deviation (mean ± SD), based on a sample size of *n* = 3.

The other detailed experimental processes were available in the Supporting Information.

## Conflicts of Interest

The authors declare no conflicts of interest.

## Supporting information




**Supporting File**: advs74093‐sup‐0001‐SuppMat.doc.

## Data Availability

The data that support the findings of this study are available from the cor‐responding author upon reasonable request.
