# Supplementary material for: Synergistic Intra‐ and Inter‐Nanozyme Electron Transfer through Interfacial Assembly for Enhanced Multi‐Enzyme Activity
Source: Adv Sci (Weinh). 2026 Jan 28;13(19):e24274. doi: 10.1002/advs.202524274 (PMC13045441; doi:10.1002/advs.202524274)
Supplement: Supplementary file 1 — Supporting File: advs74093‐sup‐0001‐SuppMat.doc. [file ADVS-13-e24274-s001.doc]

**Supporting Information for**

**Synergistic Intra- and Inter-Nanozyme Electron Transfer Through Interfacial Assembly for Enhanced** **Multi-Enzyme Activity**

*Kun Lu^1^, Jizi Liu^2^, Xiaoyang Zhu^1^, Yu Mao^2^, Hongliang He^1^, Pingqiang Cai^3^*, Yan Li^1^*, Ning Gu^1, 2^**

1. Jiangsu Key Laboratory for Biomaterials and Devices, School of Biological Science and Medical Engineering, Southeast University, Nanjing 210009, P. R. China

2. Jiangsu Key Laboratory for Cardiovascular Information and Health Engineering Medicine, Nanjing Research Center for Biomedical Electron Microscopy, Institute of Clinical Medicine, Nanjing Drum Tower Hospital, Medical School, Nanjing University, Nanjing 210093, P. R. China

3. Jiangsu Key Laboratory of Molecular Medicine, Medical School, Nanjing University, Nanjing 210093, P. R. China

To whom correspondence may be addressed.

E-mail: pqcai@nju.edu.cn, liyan@seu.edu.cn, guning@nju.edu.cn

**Table of Contents**

Supplemental Experimental Procedures………………2

Supplemental Figures…………8

Supplemental Tables……………39

Supplemental References……………40

1. **Supplemental Experimental Procedures**

**1.1 Reagents and Instruments**

Ammonium heptamolybdate, Thiourea, Potassium ferrocyanide (K₄[Fe(CN)₆]), Ferric chloride (FeCl_3_), Polyvinylpyrrolidone (SPVP), 3,3’,5,5’-tetramethylbanzidine (TMB), 5,5’-dithiobis-(2-nitrobenzoic acid) (DTNB), Methionine, Riboflavin, Nitrotetrazolyl Blue, S-Nitrosoglutathione, 2-Hydroxybenzoic acid, p-Aminobenzenesulfonic acid and Nitrite. All reagents were commercially available and used without further purification. Ultrapure water (18.2 MΩ/cm) was used in the synthetic experiments.

Transmission electron microscopy (TEM) images were recorded on a Titan G2 60-300 transmission electron microscope at an accelerating voltage of 300 KV. Powder X-ray diffraction (XRD) patterns were collected on SmartLab SE Rigaku using Cu Kα radiation (λ=1.5418 Å). FT-IR measurements were performed using a Thermo Scientific IS5. Raman spectra were obtained by HORIBA Scientific LabRAM HR Evolution with a laser excitation wavelength of 532 nm. A dynamic light scattering instrument Zetasizer Nano ZS90 was selected to measure the Zeta potential. UV-vis spectra and solid UV diffuse reflectance spectra were obtained by Shimadzu UV3600IPLUS, where barium sulfate was selected as a reference for solid UV diffuse reflectance spectra. X-ray photoelectron spectroscopy (XPS) were collected using a Thermo Scientific ESCALAB 250Xi X-ray photoelectron spectrometer. Ultraviolet photo-electron spectroscopy (UPS) were collected using the Thermo Fisher ESCALAB Xi+.

**1.2 Density Functional Theory**

The study used Vienna Ab-initio Simulation Package (VASP) to perform density functional theory calculations with projector augmented wave.^[1]^ The spin polarization effect was considered in all calculation. The Perdew-Burke-Ernzerhof functional was employed for exchange-correlation effects, while DFT+D3 was used for handling weak interactions.^[2]^ The cut-off energy for the plane-wave basis was 450 eV. K-points were 2*2*1 in the Brillouin zone for structure relaxation and 3*3*1 for self-consistent calculation. 15 Å of layer vacuum was applied at Z-axis of slab models to avoid the Periodic effect. Energy and maximum stress were converged to 10^-5^ eV and 0.02 eV/Å, respectively.

**1.3 Simulated Enzyme Detection Method**

In order to more accurately compare and detect enzyme-like activities, the MoS_2_ content was selected as the quantitative standard for the enzyme activity detection in this experiment, that is, the MoS_2_ in the same comparison experimental group and the MoS_2_/PB have the same mass of MoS_2_. At the same time, the corresponding PB is the PB content loaded with the same mass. The specific detection method is as follows.

**1.3.1 Catalase (CAT)-like Assay**

CAT is an enzyme that can catalyze the decomposition of hydrogen peroxide into water and oxygen. Its enzymatic activity is one of the key properties of the antioxidant defense system in organisms. According to previous work, under weak acid/neutral conditions, PB can catalyze the disproportionation reaction of H_2_O_2_ to produce H_2_O and O_2_, showing CAT-like properties.^[3,4]^ Therefore, the use of a dissolved oxygen meter to detect the concentration of O_2_ produced by NPs catalyzing H_2_O_2_ in a fixed container can indirectly express the activity intensity of CAT. In this paper, Phosphate buffered saline (PBS 1×) was selected as the reaction environment, and a certain volume of H_2_O_2_ and a certain volume of nanozyme solution of different concentrations were added in turn. After adding, the measurement can be started. Among them, the concentration of H_2_O_2_ in the detection system is 0.025 M.

**1.3.2 Peroxidase (POD)-like Assay**

POD uses H_2_O_2_ as an oxidant to catalyze the oxidation reaction of other substances and reduce H_2_O_2_ to water (H_2_O), thereby removing excess H_2_O_2_ in the cell. According to previous work, PB can catalyze H_2_O_2_ to oxidize colorless TMB to a blue TMBox product under acidic conditions (pH ≈ 4), showing strong POD-like enzyme activity.^[5,6]^ Specifically, TMBox has a maximum absorption peak at 650 nm, so the POD enzyme performance can be reflected by detecting the absorption spectrum of the solution in the range of 500-750 nm. Specifically, a 0.2 M sodium acetate solution is configured as the reaction microenvironment, and a certain amount of H_2_O_2_, TMB, and nanozyme solutions of different concentrations are added to the reaction system in sequence and then detected at fixed time points. (TMB 2 mg/mL). The dynamic testing method is consistent with our team's previous research. (TMB 10 mg/mL).^[7,8]^

**1.3.3 Superoxide Dismutase (SOD)-like Enzyme Detection**

SOD is a class of enzymes that can catalyze the dismutation of superoxide anion free radicals (O_2_^·−^) into H_2_O_2_ and O_2_. According to previous work reports, the methionine-riboflavin-NBT photoreduction method can be used to detect whether NPs have SOD-like activity.^[9]^ Specifically, riboflavin reduced by methionine reacts with O_2_ to produce O_2_^·−^, which can reduce the slightly yellow NBT to blue formazan with a maximum absorption peak at 560 nm. SOD can inhibit the formation of blue formazan by catalyzing the dismutation reaction of O_2_^·−^ to produce O_2_ and H_2_O_2_. Therefore, after the photoreduction reaction, the higher the SOD-like enzyme activity, the lower the absorption peak of its reaction solution at 560 nm. At the same time, in order to avoid the influence of the absorption intensity of NPs, the absorption intensity of the corresponding concentration of ultraviolet specific wavelength will be deducted from each experimental group. In the specific implementation of this article, phosphate buffered saline (PBS 1×) was still selected as the reaction environment. Specific concentrations of methionine, riboflavin, NBT, EDTA, and the corresponding nanozyme solutions were added in sequence and then detected at fixed time points.

**1.3.4 Glutathione Peroxidase Performance Test**

Glutathione peroxidase (GSH-Px) is an important peroxide decomposing enzyme that can catalyze the conversion of GSH into GSSG, reducing toxic peroxides to non-toxic hydroxyl compounds. Usually, this type of enzymatic activity can be detected by measuring the content of GSH.^[10]^ Therefore, Ellman's reagent (5,5-dithiobis(nitrophenol), DTNB) can be used for detection.^[11]^ Specifically, DTNB can react with GSH to generate a yellow product, TNB, which can indirectly reflect the GSH content through the change in the absorbance of the reaction solution at 412 nm. In short, the smaller the absorbance of the reaction solution at 412 nm, the greater the GSH-Px activity.

**1.3.5 S-Nitrosoglutathione (GSNO) Reductase Performance Test**

Controlling the production of nitric oxide (NO) from endogenous sources such as S-nitrosoglutathione (GSNO) is of great significance to biomedicine.^[12]^ According to relevant literature reports, some inorganic nanozymes can promote the conversion of GSNO to NO.^[13]^ Therefore, the purple or pink azo compound product obtained by the reaction of Gress reagent with NO can be used to detect the concentration of NO produced by observing the change in its absorption peak at 540 nm. This indirectly reflects the effect of nanozymes on the promotion of GSNO to NO production, that is, the higher the absorption peak, the greater the amount of NO produced, and the stronger its GSNO reductase performance. Since GSNO will self-decompose under visible light, this detection method is carried out under dark conditions.

**1.3.6 Nitrite Reducing Enzyme Activity Test**

Nitrates in the organism can be converted into NO, which can regulate a variety of cell signaling pathways, including regulating blood flow, flow rate, and vascular resistance under physiological conditions. Therefore, it is necessary to explore whether the relevant nanozymes can regulate the conversion of nitrates into NO. According to relevant literature reports, nitrite reacts with p-aminobenzenesulfonic acid, and its intermediate product can react with α-naphthylamine to finally generate a red azo product with a maximum peak at 524 nm.^[14]^ That is, the smaller the peak, the greater the nitrite reducing enzyme activity.

**1.3.7 Hydroxyl Radical (·OH) Generation Detection**

According to current reports, iron-based nanozymes have two enzyme-like catalytic mechanisms, one is the Fenton reaction of Fe^2+^, and the other is the electron transfer mechanism. Our previous work has proved that the enzyme-like mechanism of PB is the electron transfer mechanism.^[15]^ In order to clarify the catalytic mechanism of MoS_2_/PB, it is necessary to determine whether the electron transfer mechanism is still maintained after the new structure is generated, without the interference of the Fenton reaction or Fenton-like reaction. Therefore, this paper chooses to verify the catalytic mechanism of MoS_2_/PB by using Fe^2+^ as a control. Specifically, Fe^2+^ catalyzes H_2_O_2_ to produce ·OH under acidic conditions, thereby promoting the conversion of 2-hydroxybenzoic acid into dihydroxybenzoic acid with a characteristic absorption peak in the range of 400-700 nm. The presence or absence of the absorption peak can determine whether it can have a Fenton effect or a Fenton-like effect, thereby further determining the fundamental catalytic mechanism of MoS_2_/PB.

1. **Supplemental Figures**


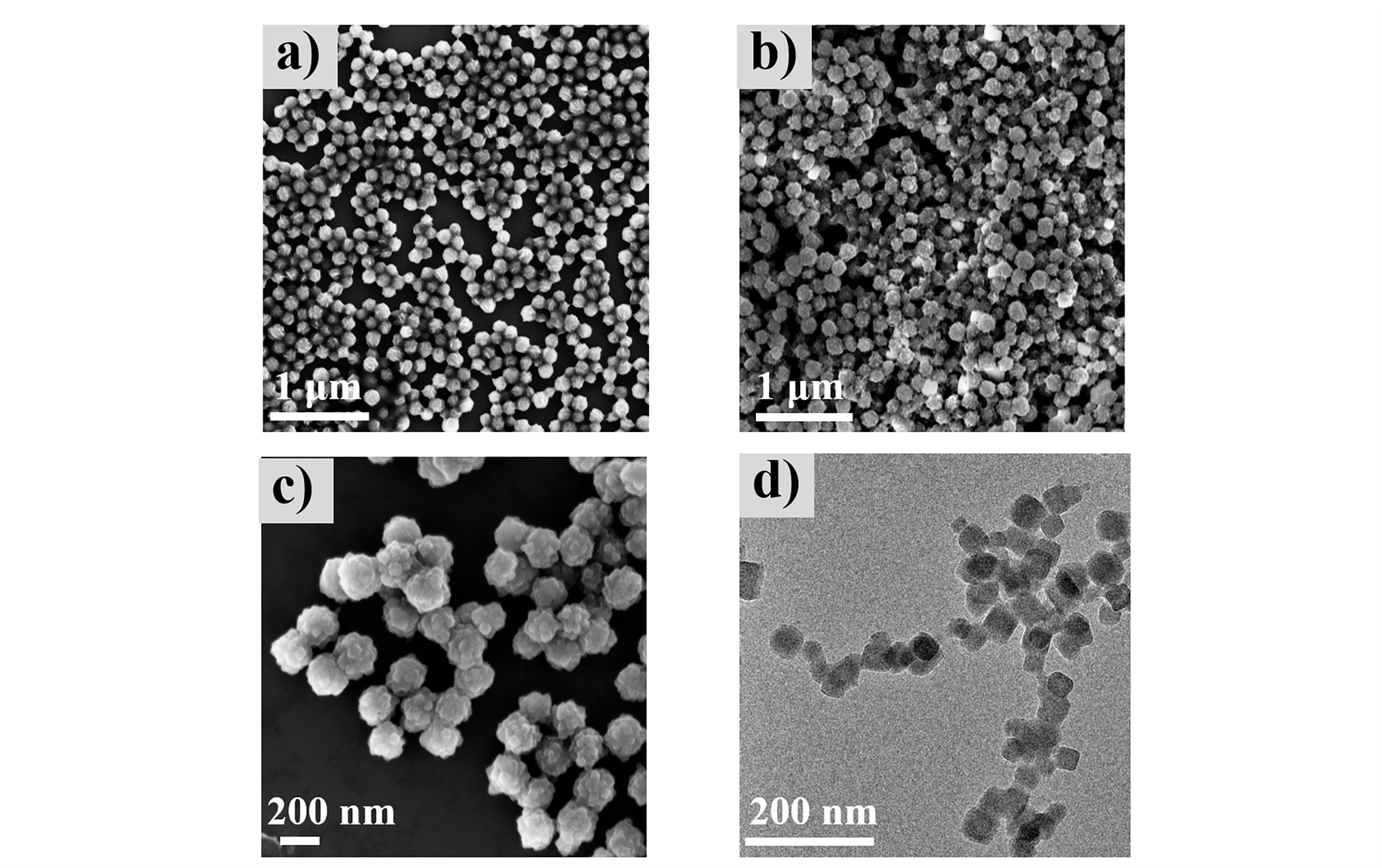


**Figure S1.** SEM images of nanozymes. (a) MoS_2_. (b) and (c) MoS_2_/PB. TEM image of nanozymes. (d) PB.


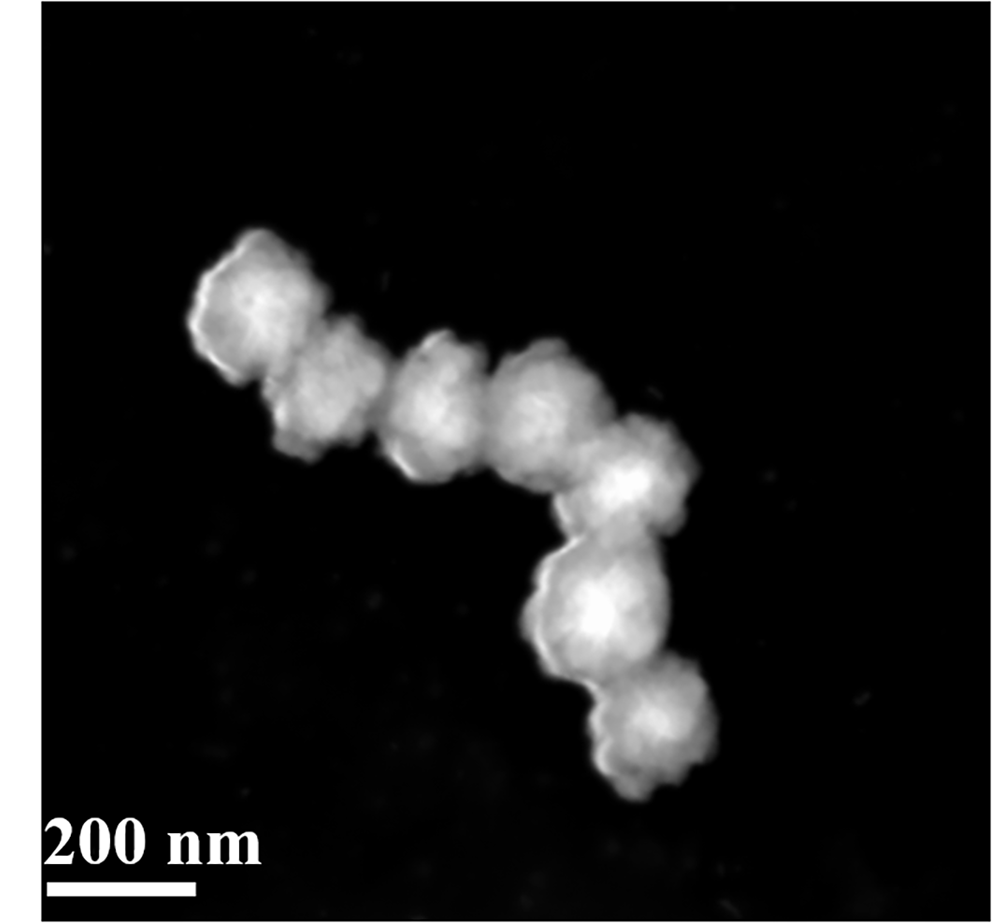


**Figure S2.** HAADF-STEM image of MoS_2_/PB.


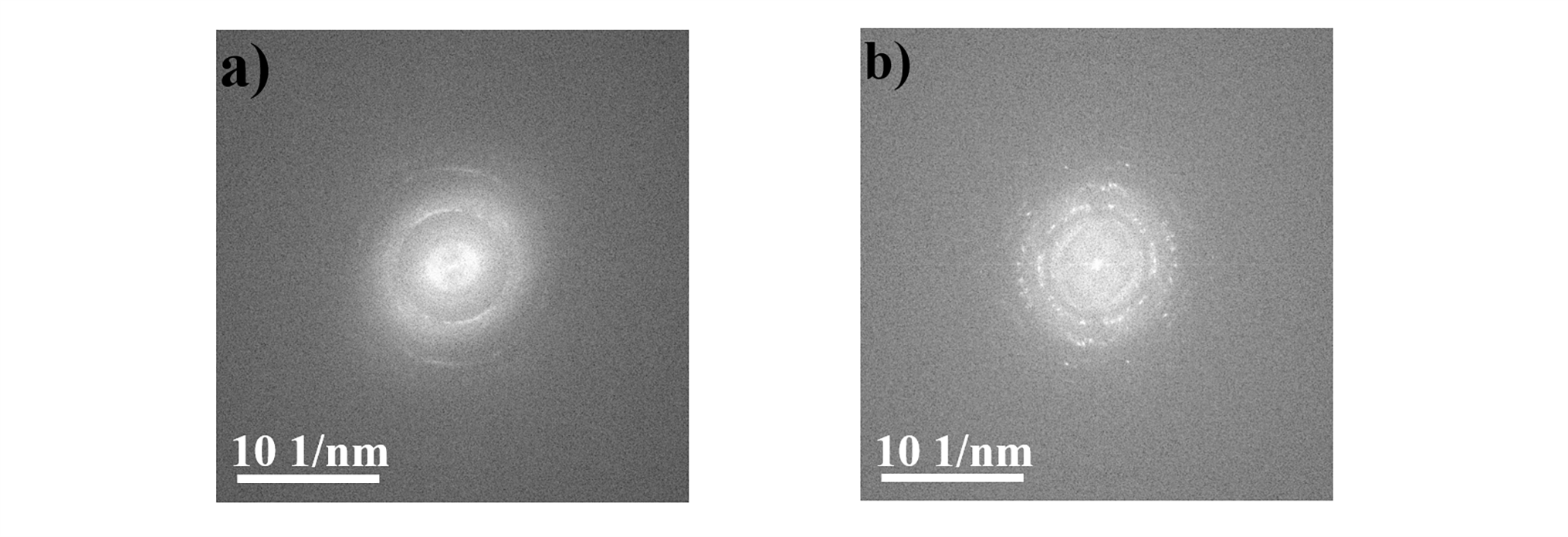


**Figure S3.** Selected area electron diffraction images obtained by Fourier transform. (a) MoS_2_. (b) MoS_2_/PB.


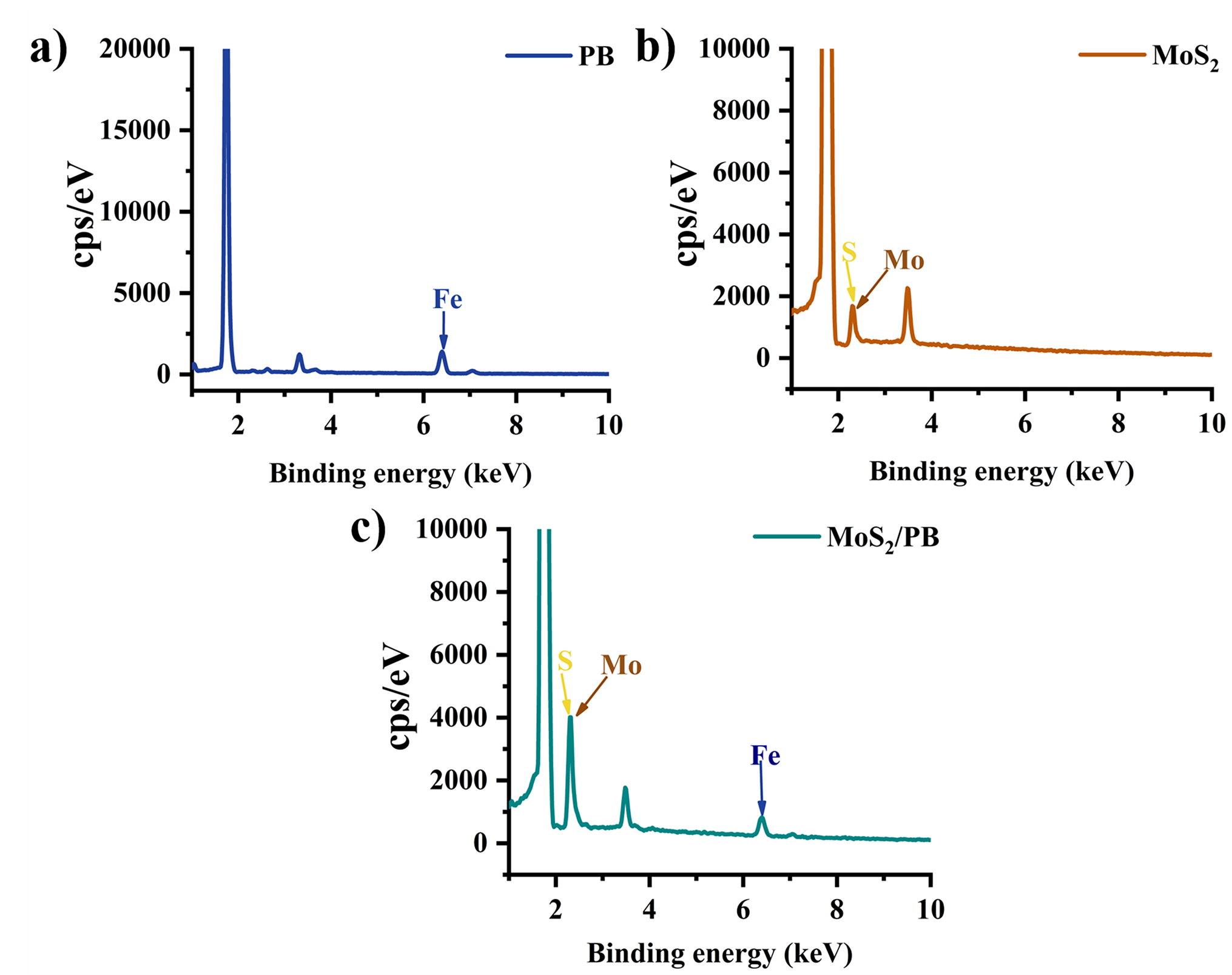


**Figure S4.** EDS spectra of nanozymes. (a) PB. (b) MoS_2_. (c) MoS_2_/PB.


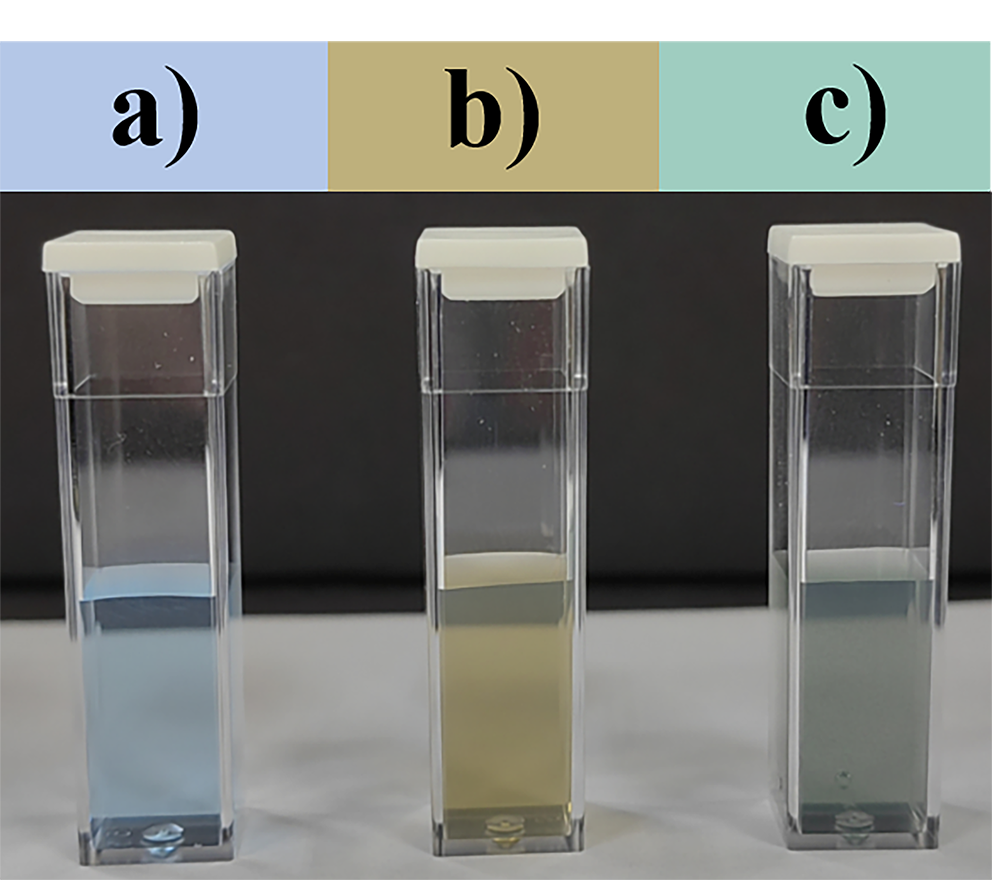


**Figure S5.** Digital image of nanozymes. (a) PB. (b) MoS_2_. (c) MoS_2_/PB.


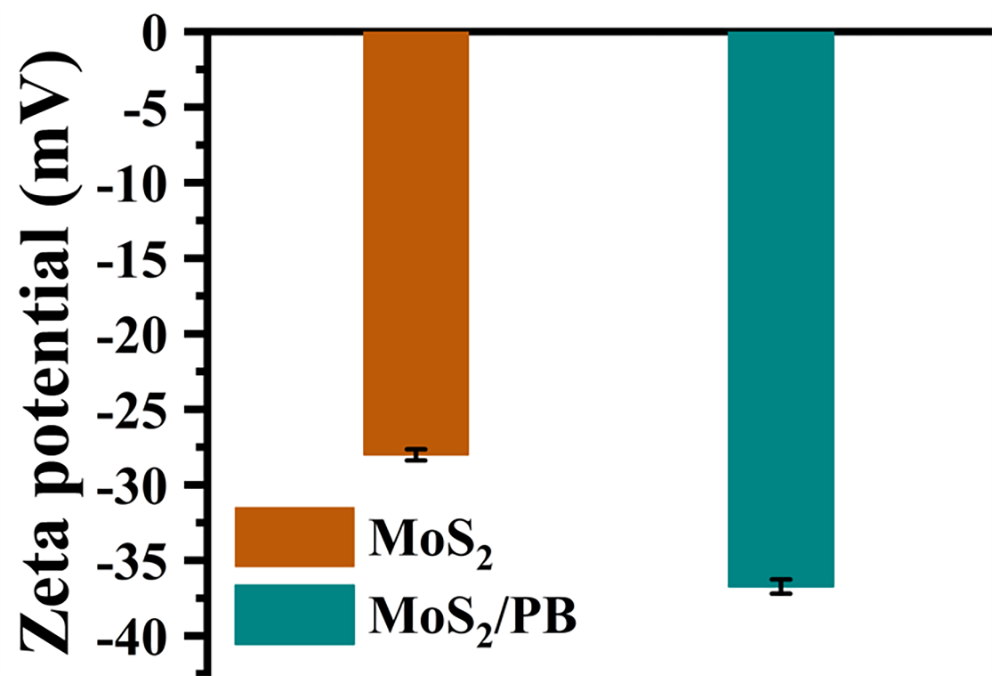


**Figure S6.** Zeta potential of MoS_2_ and MoS_2_/PB, n = 3.


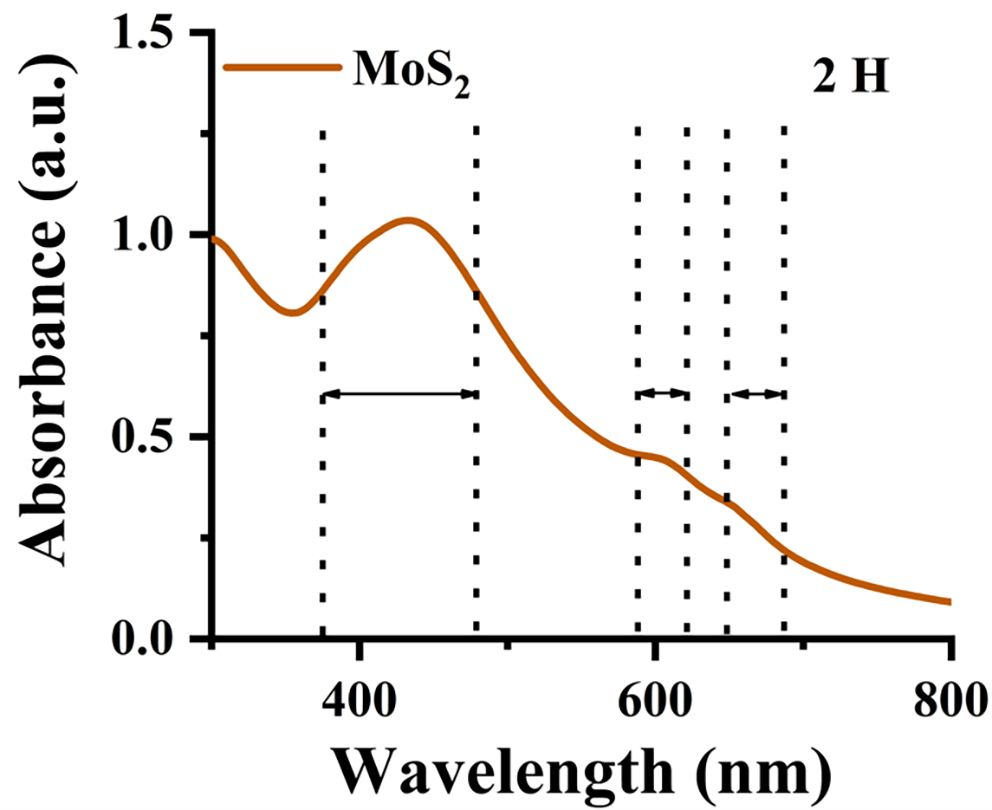


**Figure S7.** UV-Vis spectrum of MoS_2_.


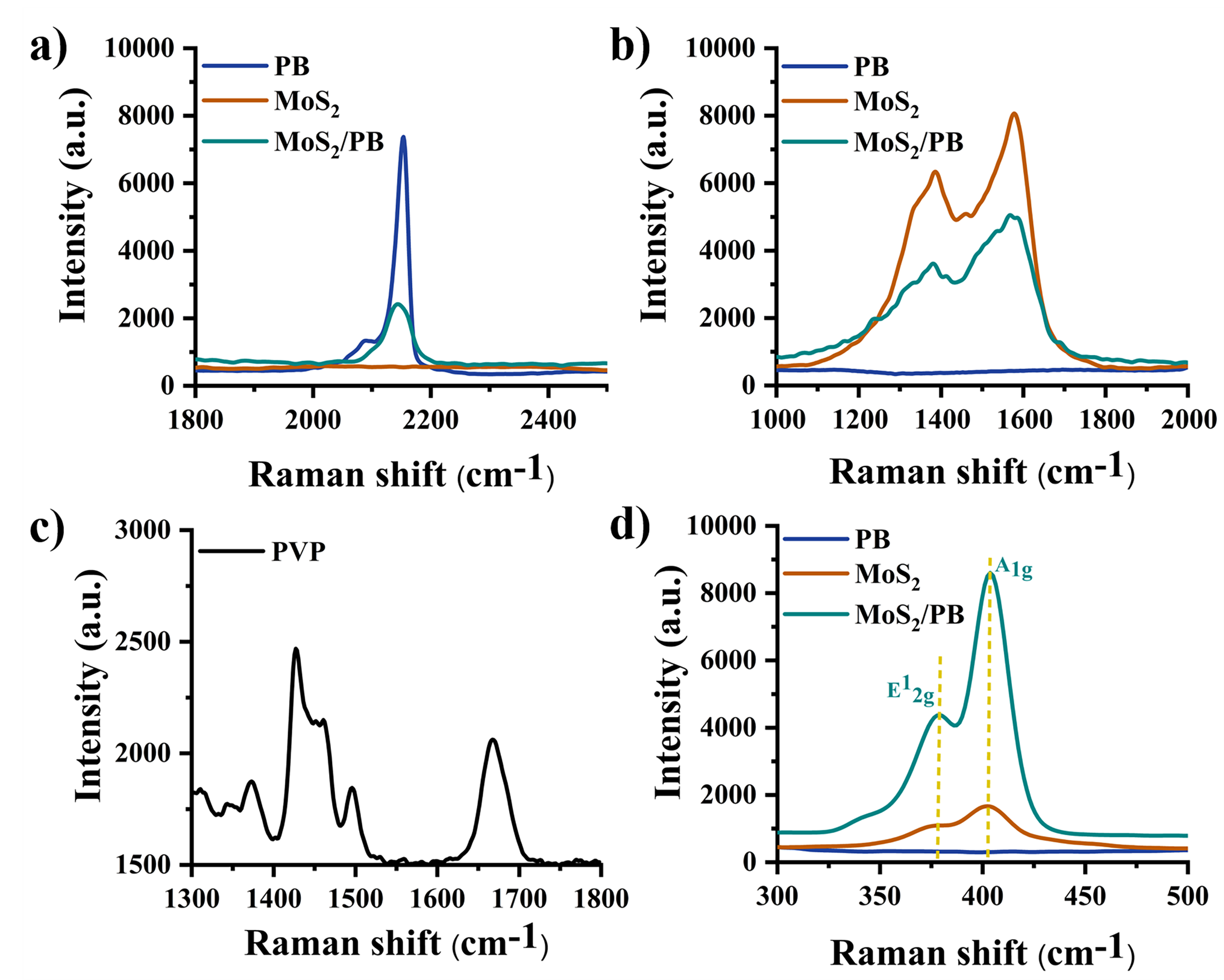


**Figure S8.** Different Raman shift regions spectra of PB, MoS_2_, MoS_2_/PB, and PVP.


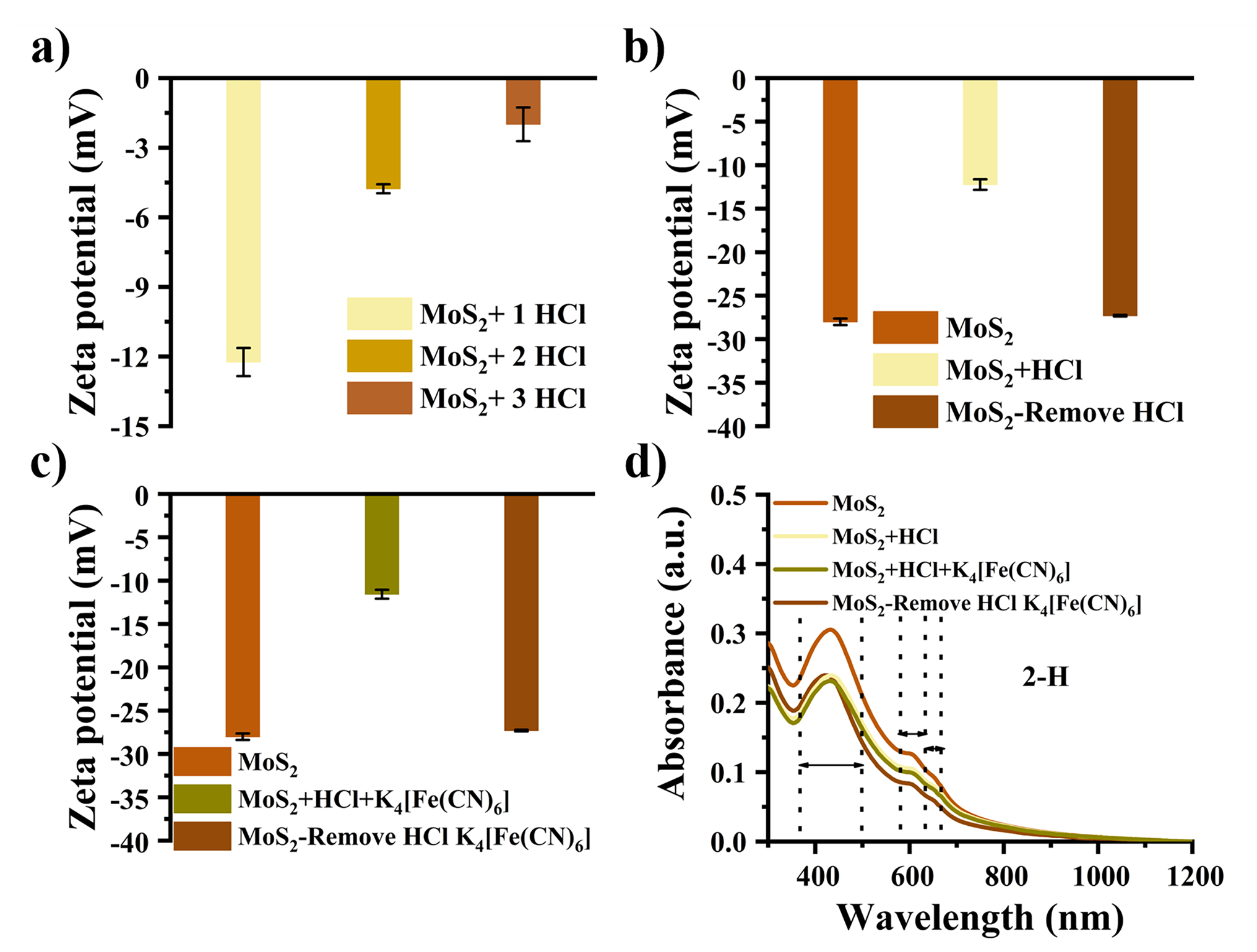


**Figure S9.** (a) Changes in the zeta potential values of MoS_2_ treated under different acidic conditions, n = 3. (b) Changes in the zeta potential values of MoS_2_ before and after treatment in acidic conditions and after removal of HCl, n = 3. (c) Changes in the zeta potential value of MoS_2_ under acidic conditions and K₄[Fe(CN)₆] treatment (MoS_2_-CN^-^), n = 3. (d) UV-Vis spectrum of MoS_2_ under different conditions.


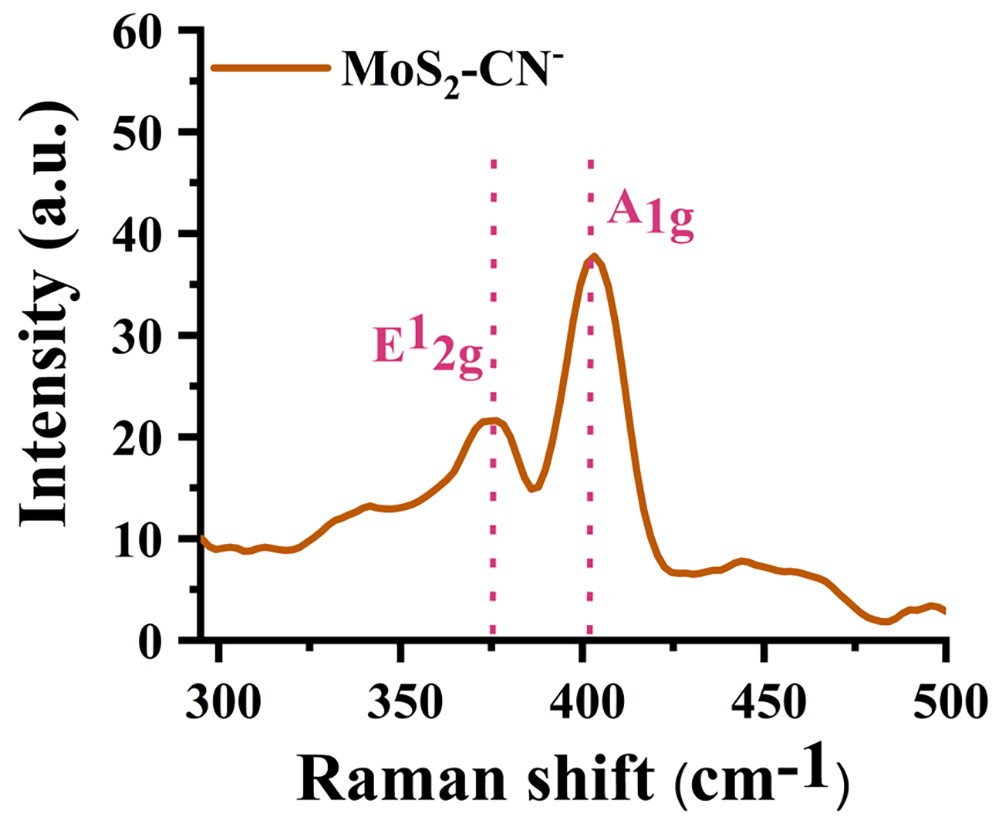


**Figure S10.** Raman spectrum of MoS_2_-CN^-^.


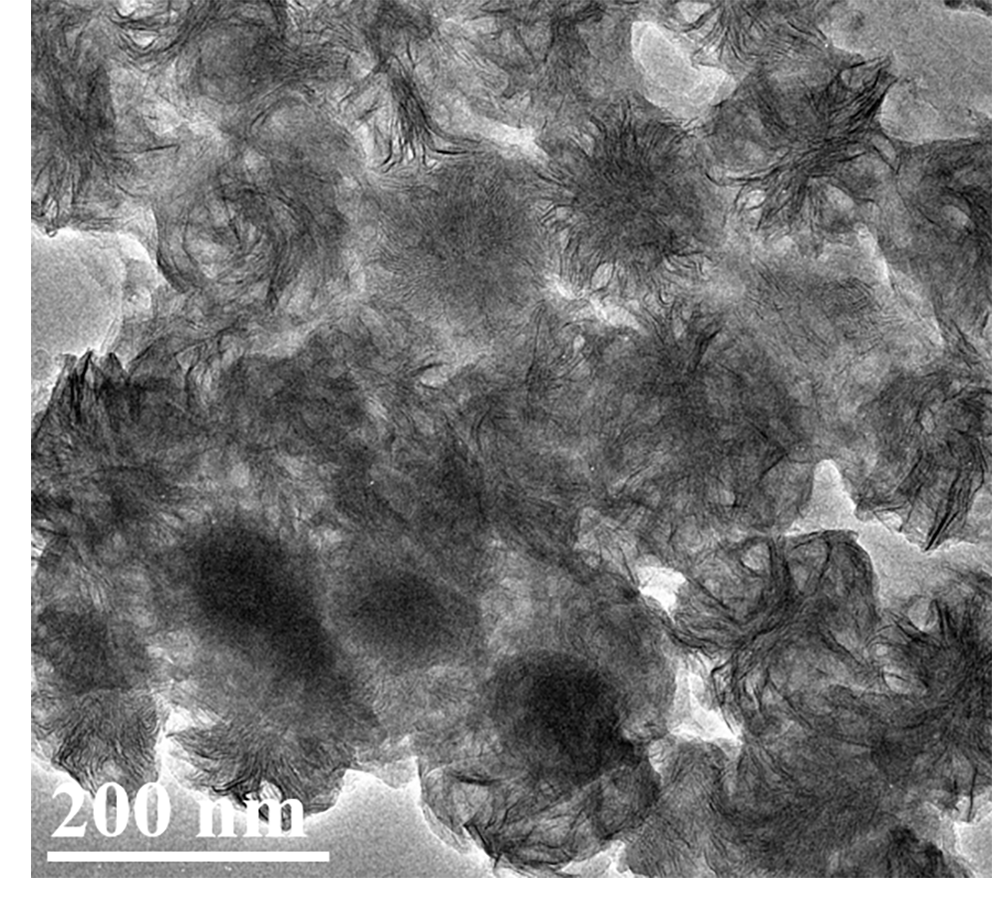


**Figure S11.** TEM image of MoS_2_-CN^-^.


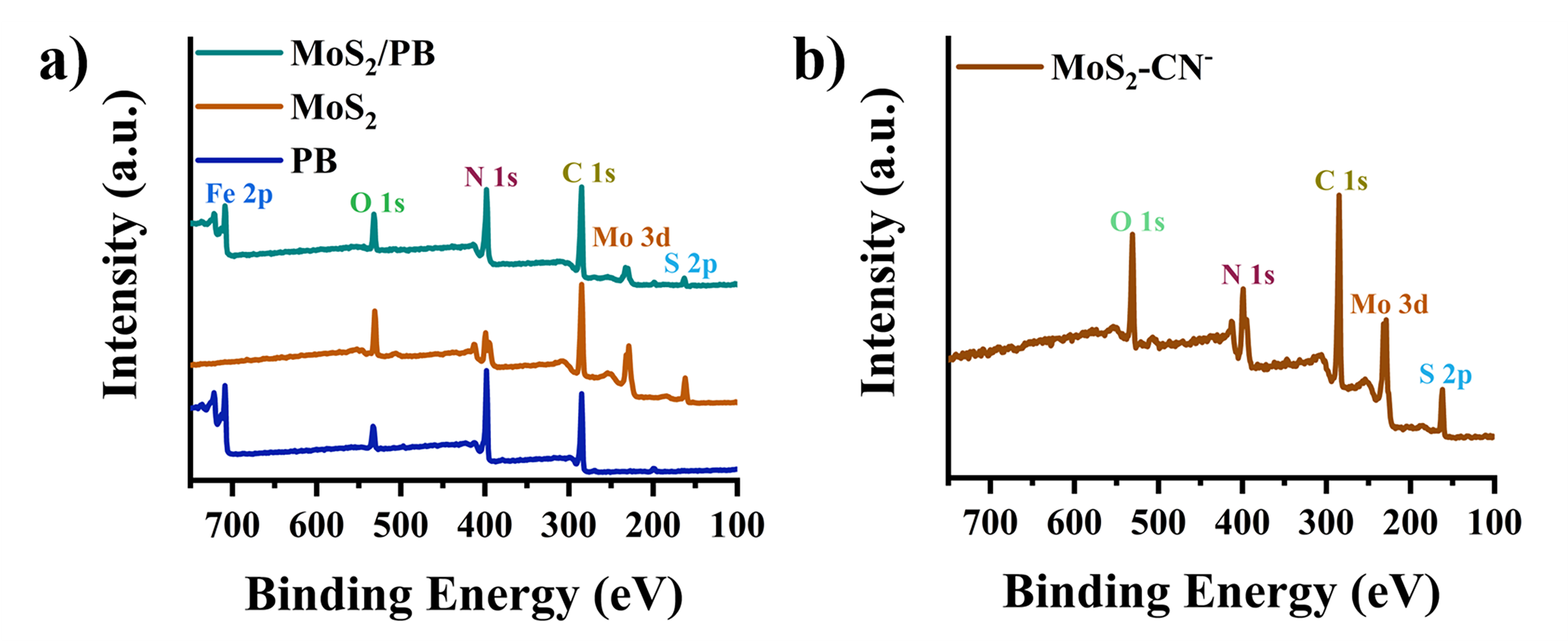


**Figure S12.** XPS survey spectra. (a) PB, MoS_2_, and MoS_2_/PB. (b) MoS_2_-CN^-^.


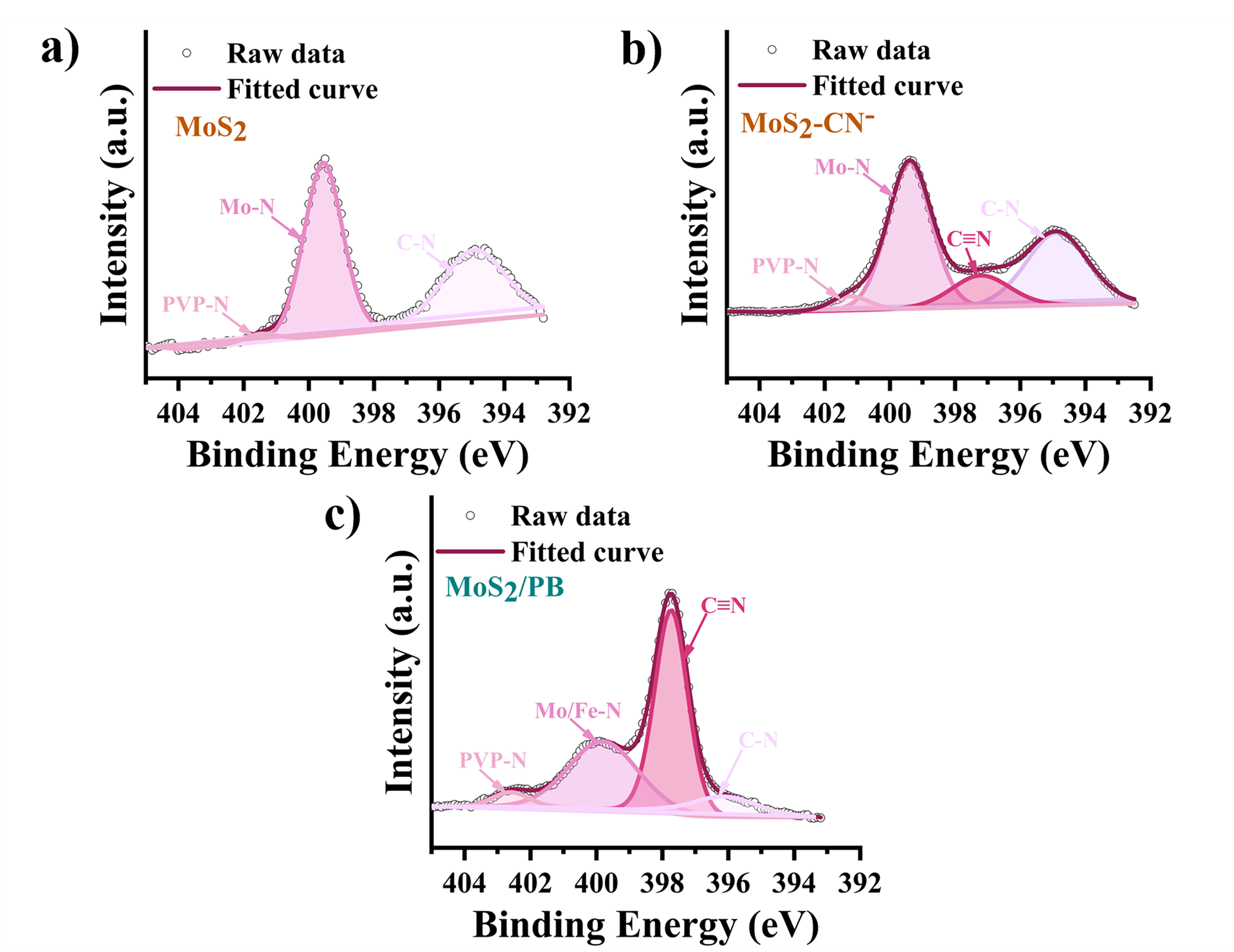


**Figure S13.** High-resolution XPS spectra of N 1s. (a) MoS_2_. (b) MoS_2_-CN^-^. (c) MoS_2_/PB.


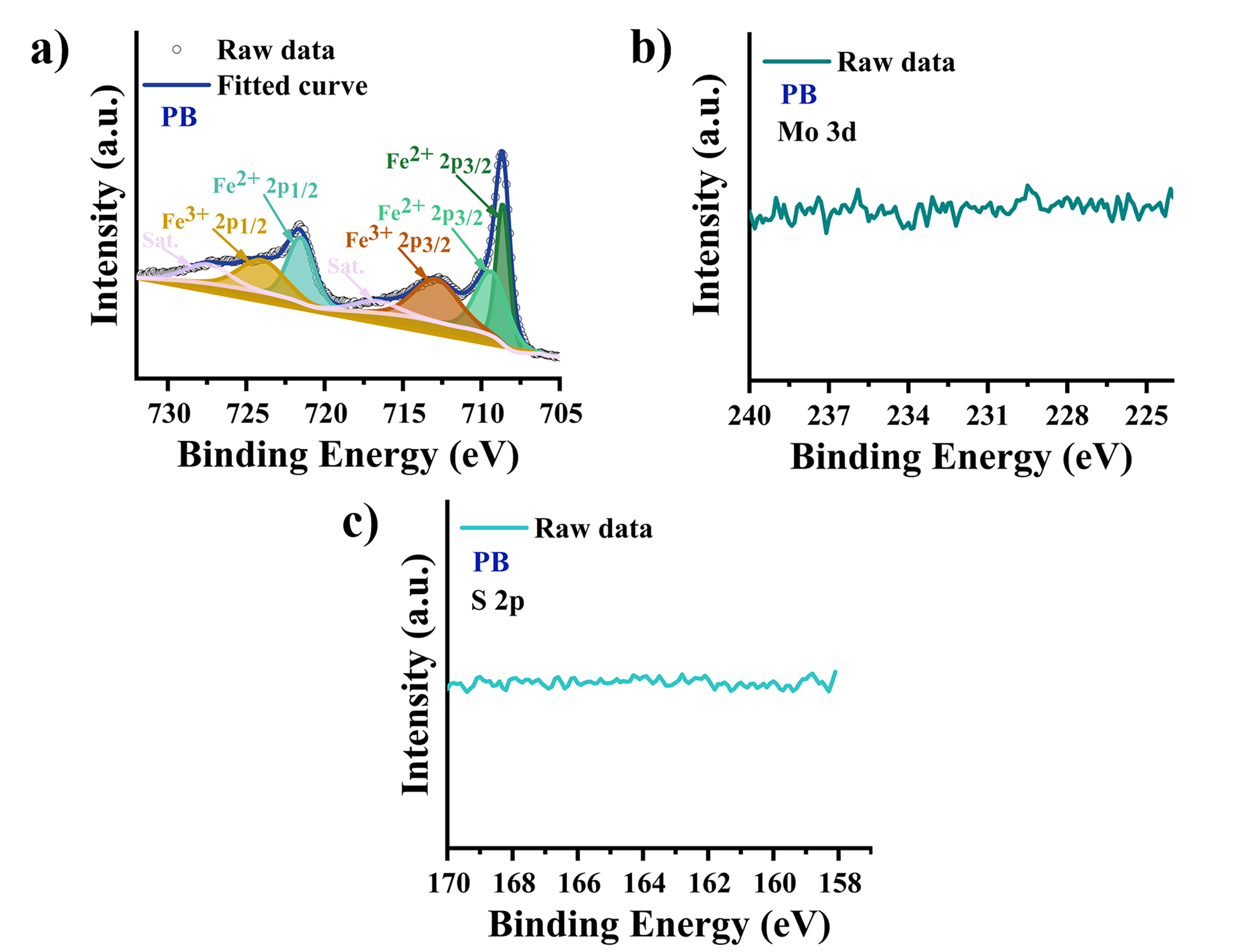


**Figure S14.** High-resolution XPS spectra of PB. (a) Fe 2p. (b) Mo 3d. (c) S 2p.


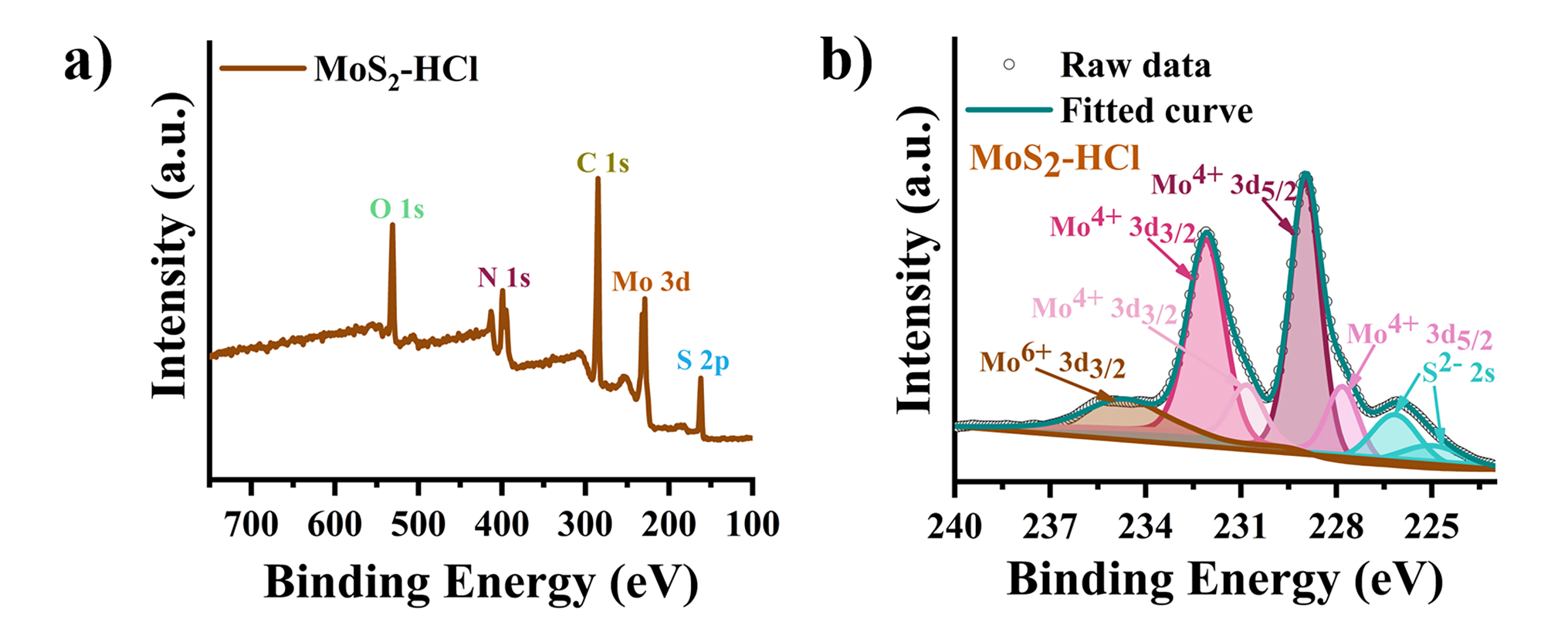


**Figure S15.** XPS spectra of MoS_2_ after HCl treatment. (a) XPS survey spectrum. (b) Mo 3d.


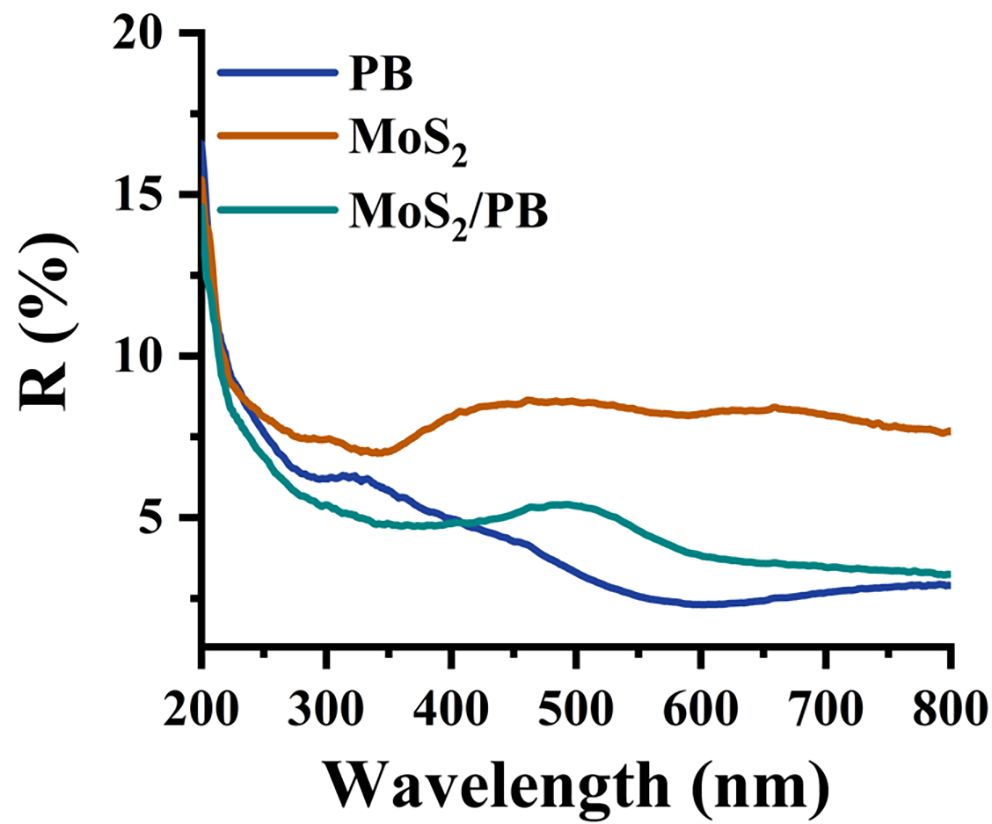


**Figure S16.** UV-Vis DRS spectrum of PB, MoS_2_, and MoS_2_/PB.


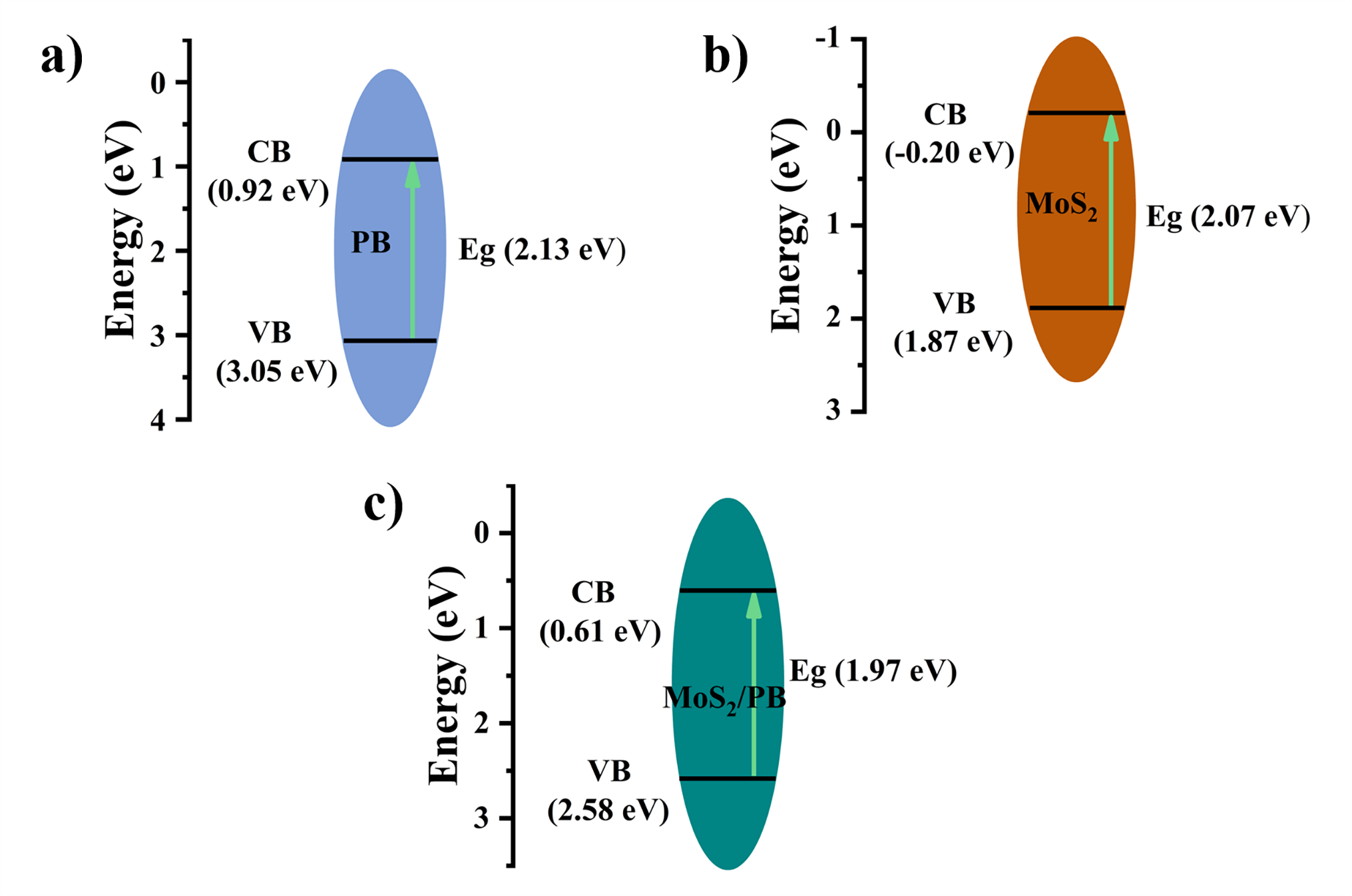


**Figure S17.** Conduction band and valence band. (a) PB. (b) MoS_2_. (c) MoS_2_/PB.

**
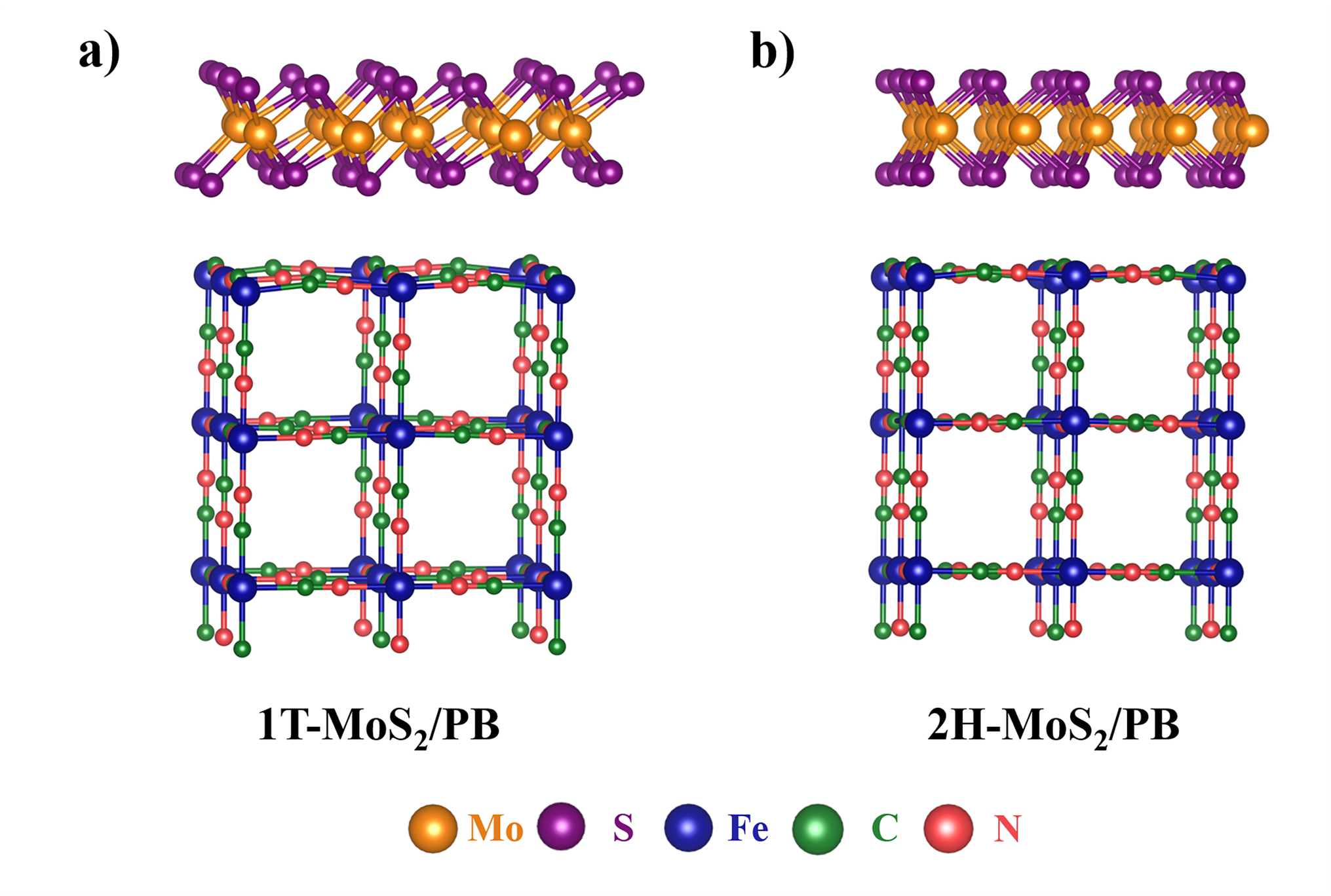
**

**Figure S18.** DFT unit cell structure model. (a) 1T-MoS_2_/PB. (b) 2H-MoS_2_/PB.


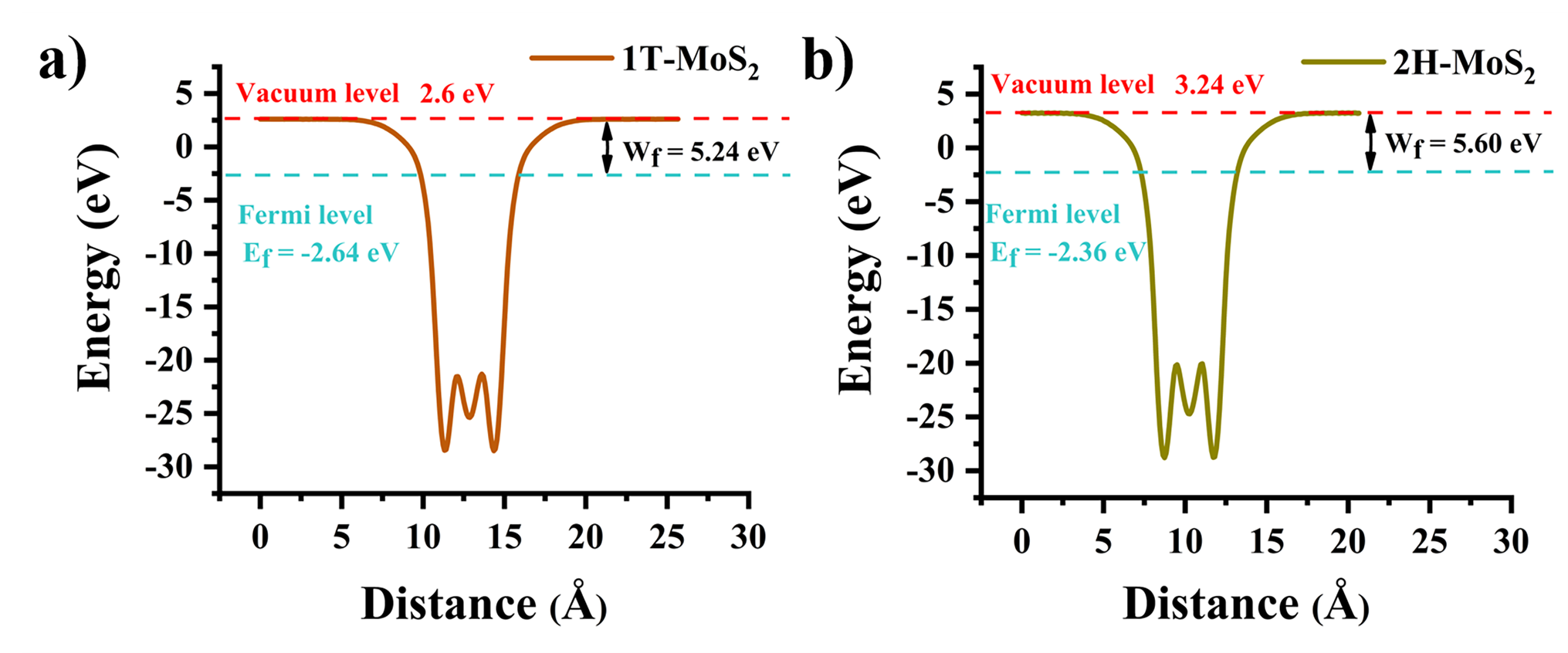


**Figure S19.** Work functions (W_f_) of different structures. (a) 1T-MoS_2_. (b) 2H-MoS_2_.


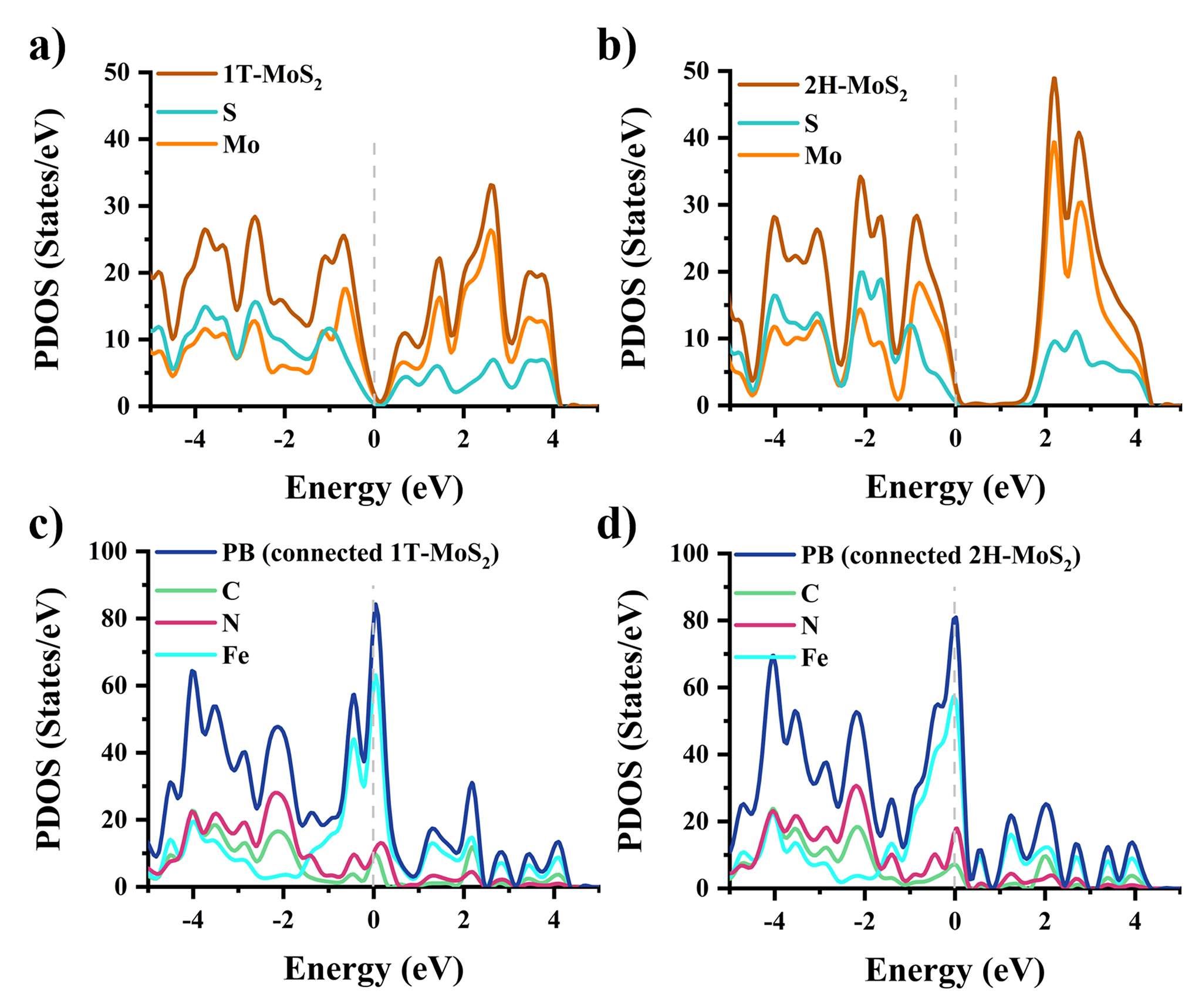


**Figure S20.** Density of states (DOS) of different structures. (a) PDOS of S and Mo in 1T-MoS_2_. (b) PDOS of S and Mo in 2H-MoS_2_. (c) PDOS of Fe, C, and N in PB connected to 1T-MoS_2_. (d) PDOS of Fe, C, and N in PB connected to 2H-MoS_2_.


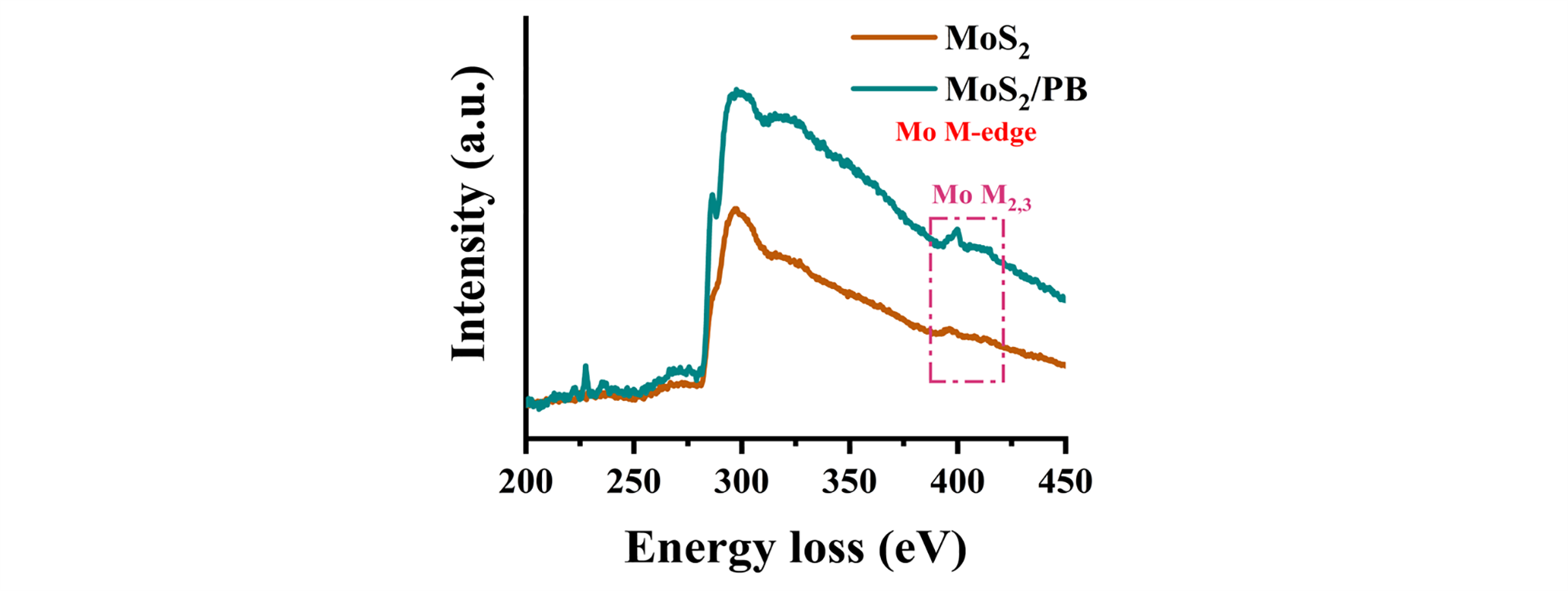


**Figure S21.** Electron energy loss spectroscopy (EELS) spectra of the Mo M-edge.


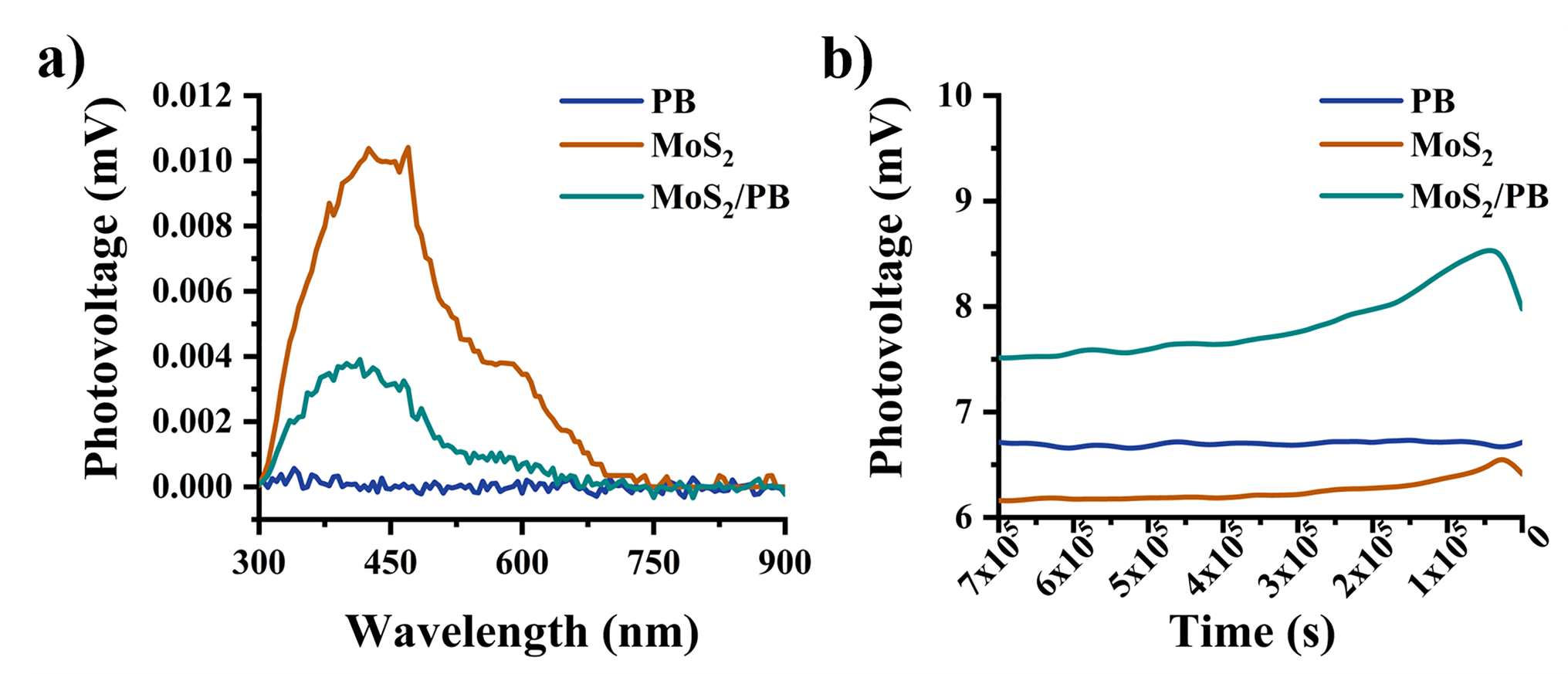


**Figure S22.** The surface photovoltage (SPV) and transient photovoltage (TPV) spectroscopy. (a) SPV. (b) TPV.


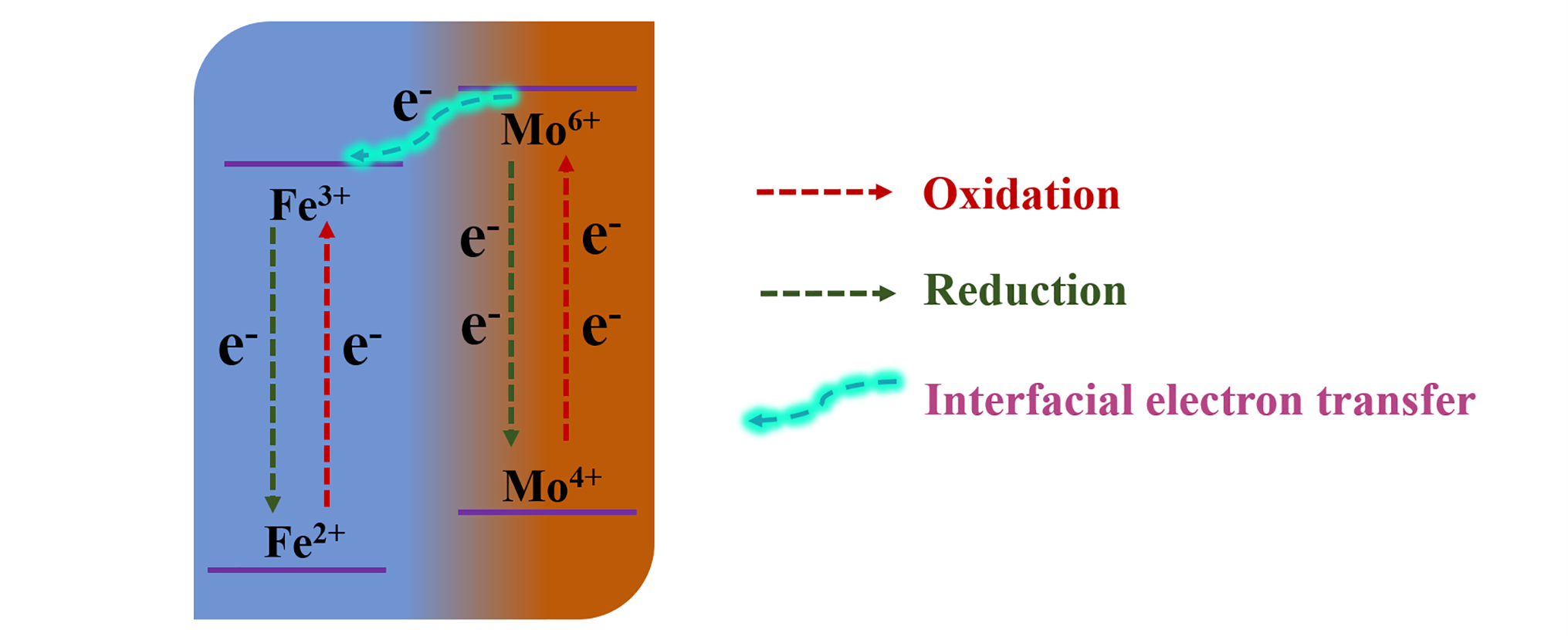


**Figure S23.** Schematic diagram of intra- and inter-nanozyme electron transfer and reduction performance improvement.


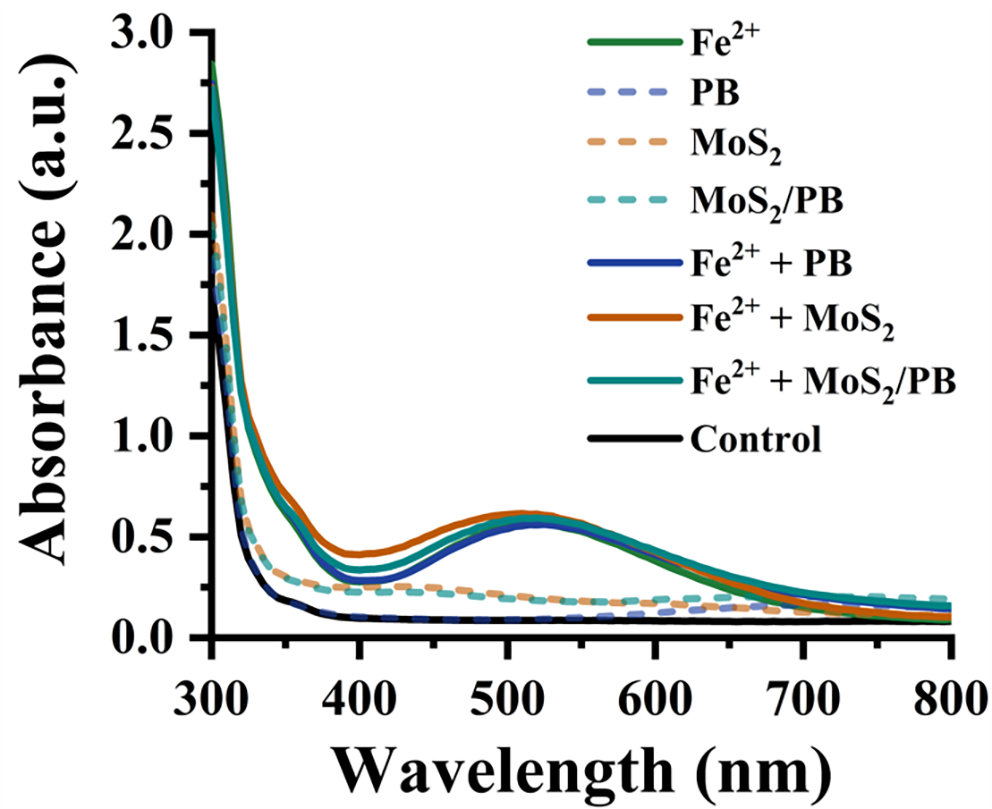


**Figure S24.** Comparison of hydroxyl radical production.


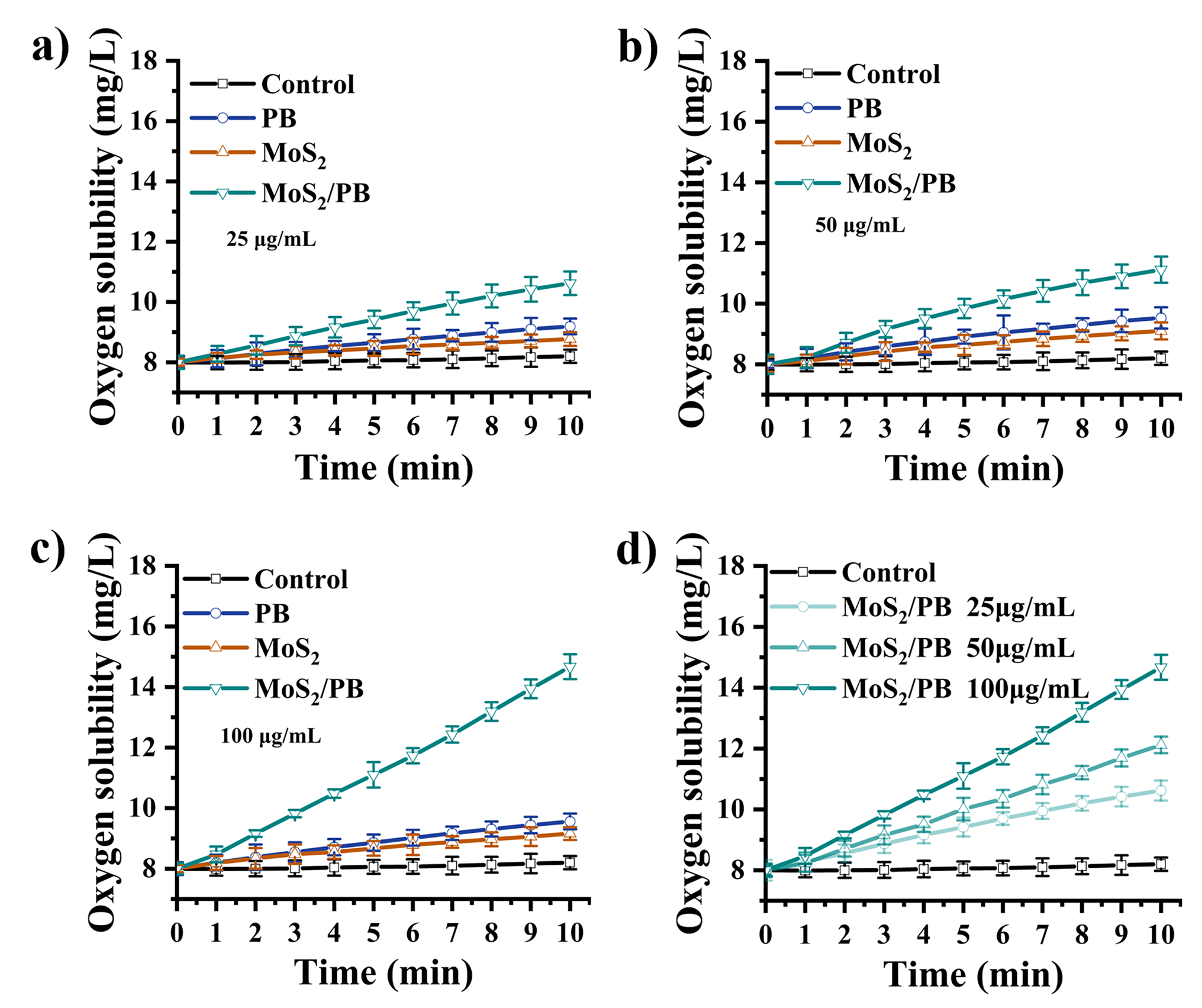


**Figure S25.** CAT-like enzyme activity of PB, MoS_2_, and MoS_2_/PB, n = 3.


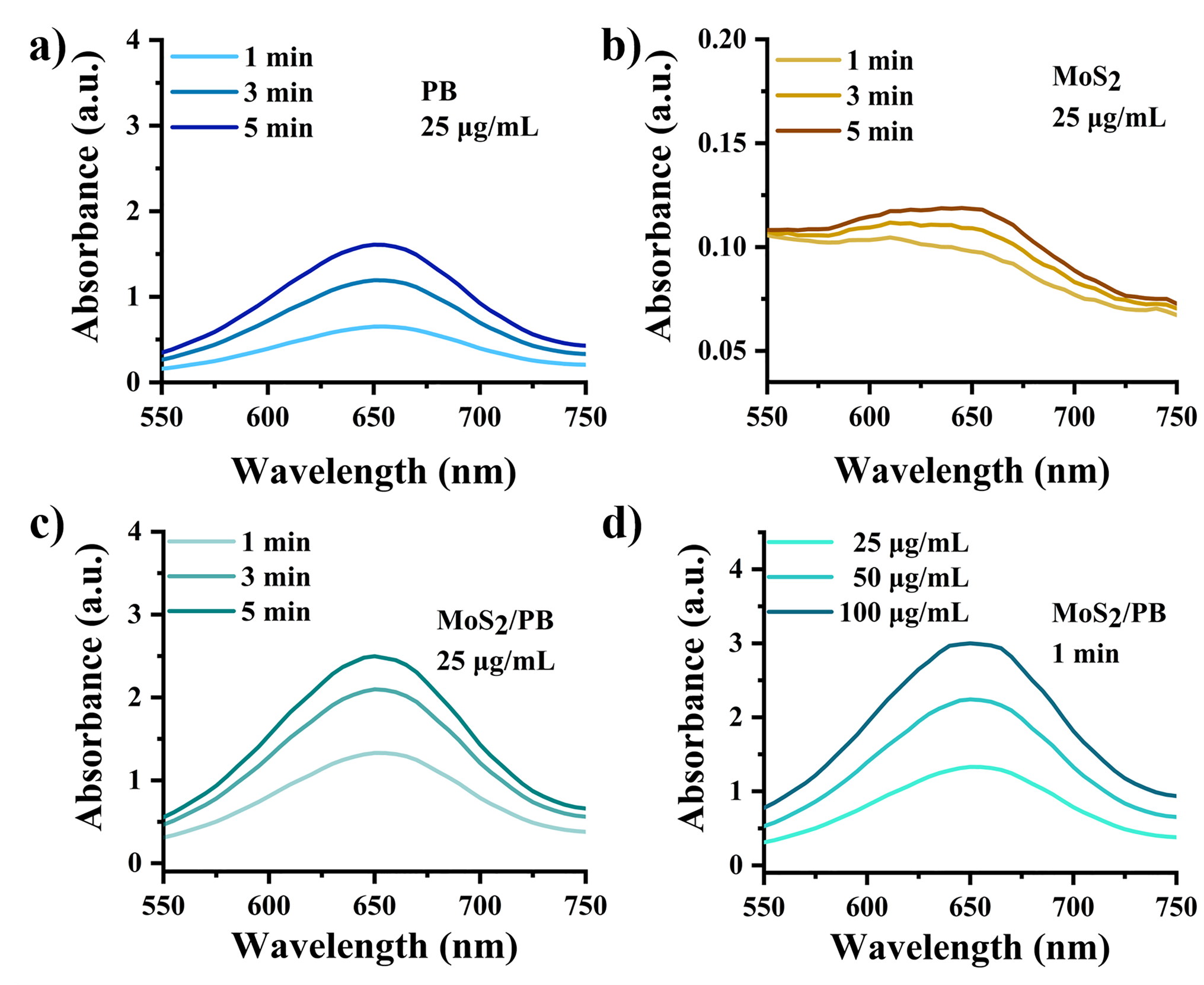


**Figure S26.** POD-like enzyme activity of PB, MoS_2_, and MoS_2_/PB. (TMB 2 mg/mL).


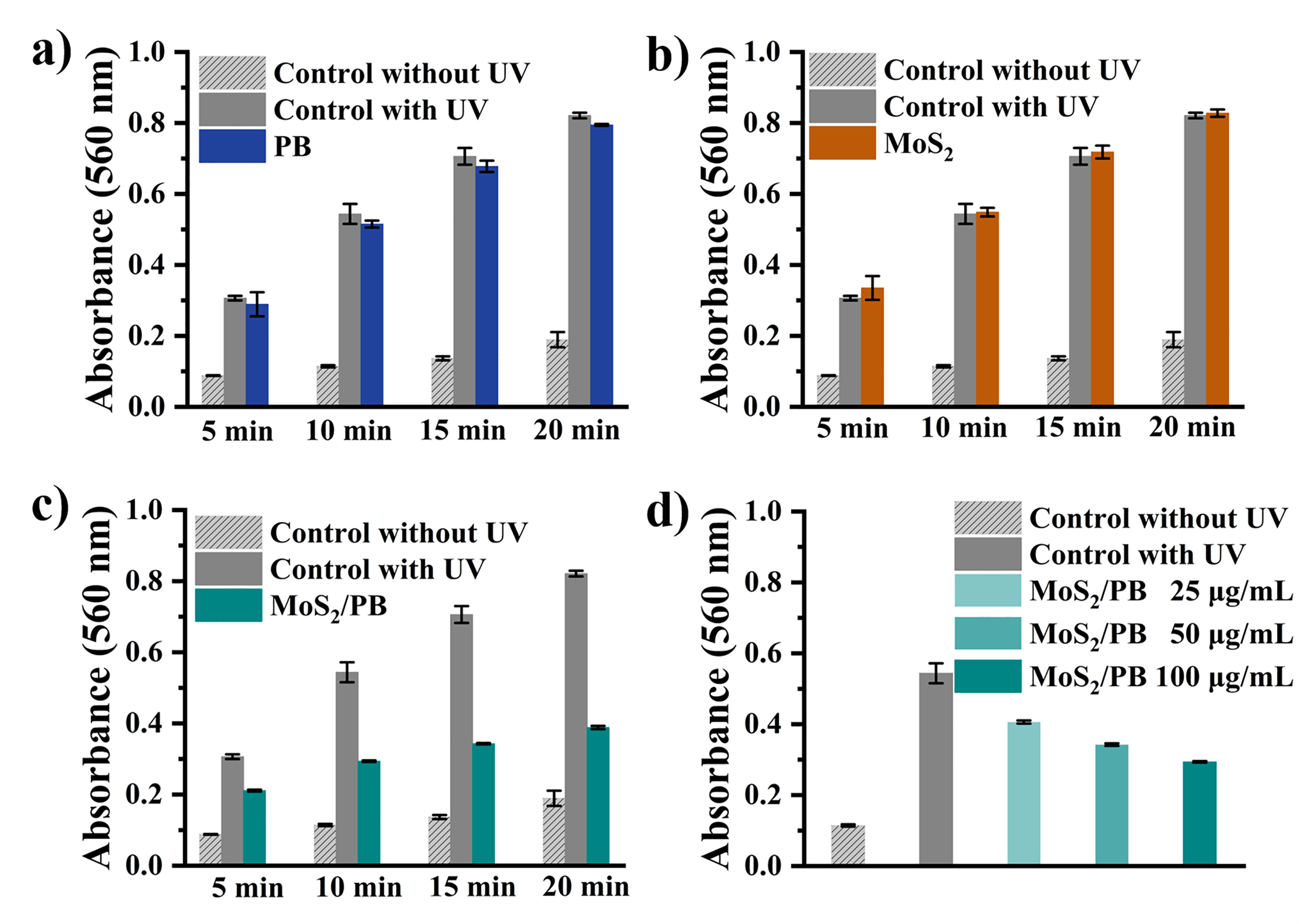


**Figure S27.** SOD-like enzyme activity of PB, MoS_2_, and MoS_2_/PB, n = 3.


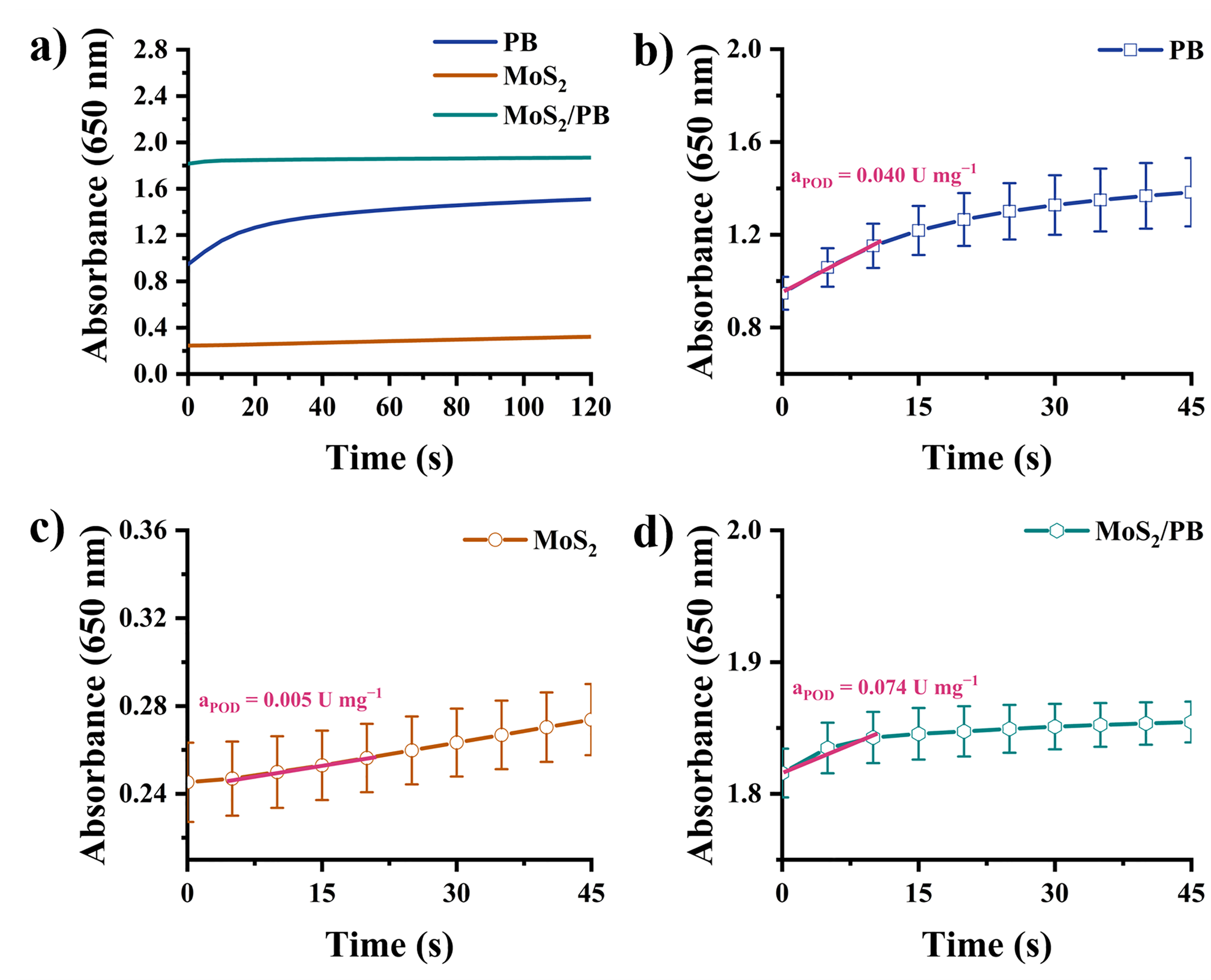


**Figure S28.** Kinetic analysis of POD-like enzyme activity for PB, MoS_2_, and MoS_2_/PB. (TMB 10 mg/mL), n = 3.


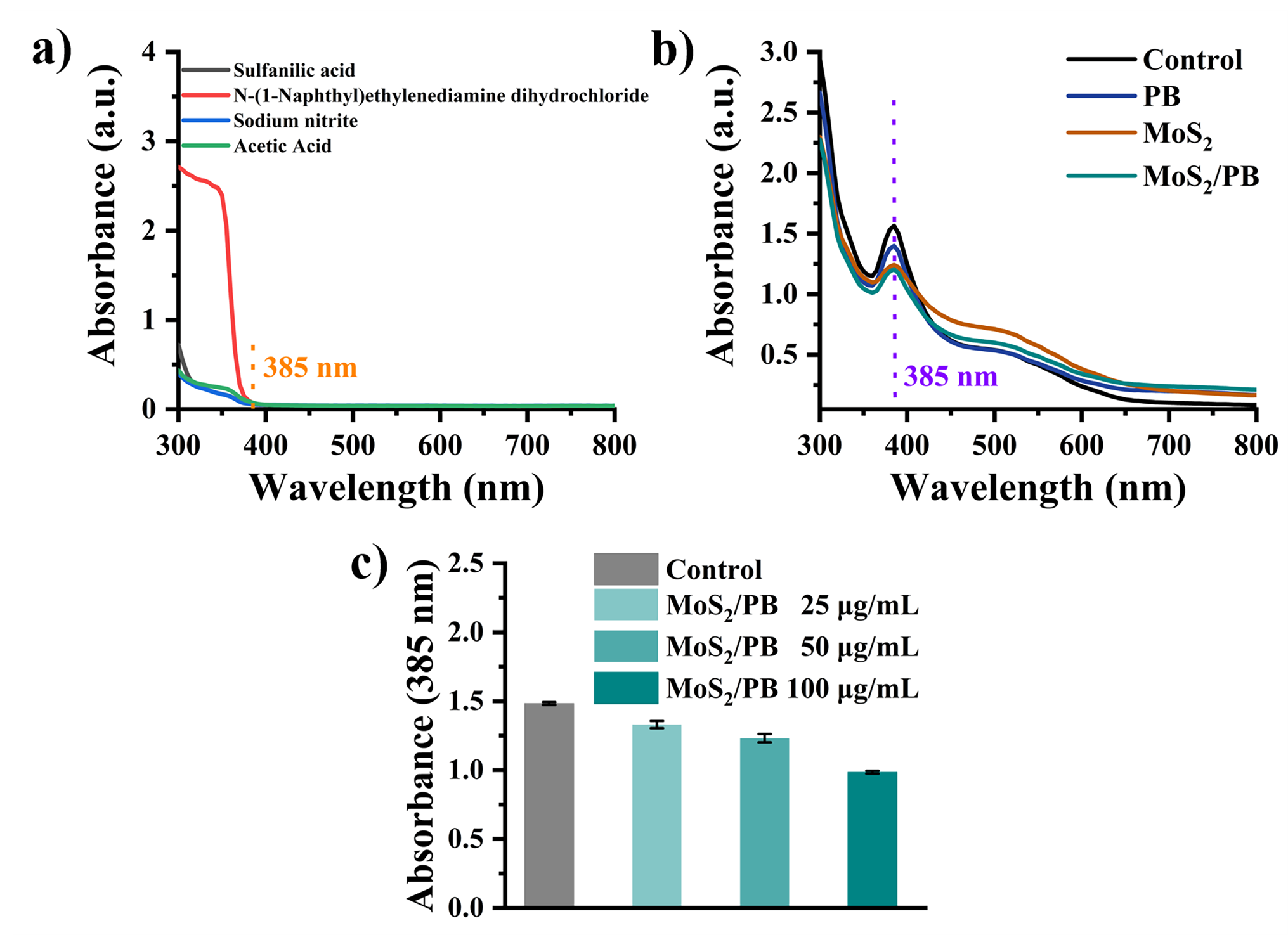


**Figure S29.** (a) UV-Vis absorption spectrum of different detection reagents. (b) UV-Vis spectrum information of NZs in NiRs test. (c) NiRs activity of MoS_2_/PB at different concentrations, n = 3.


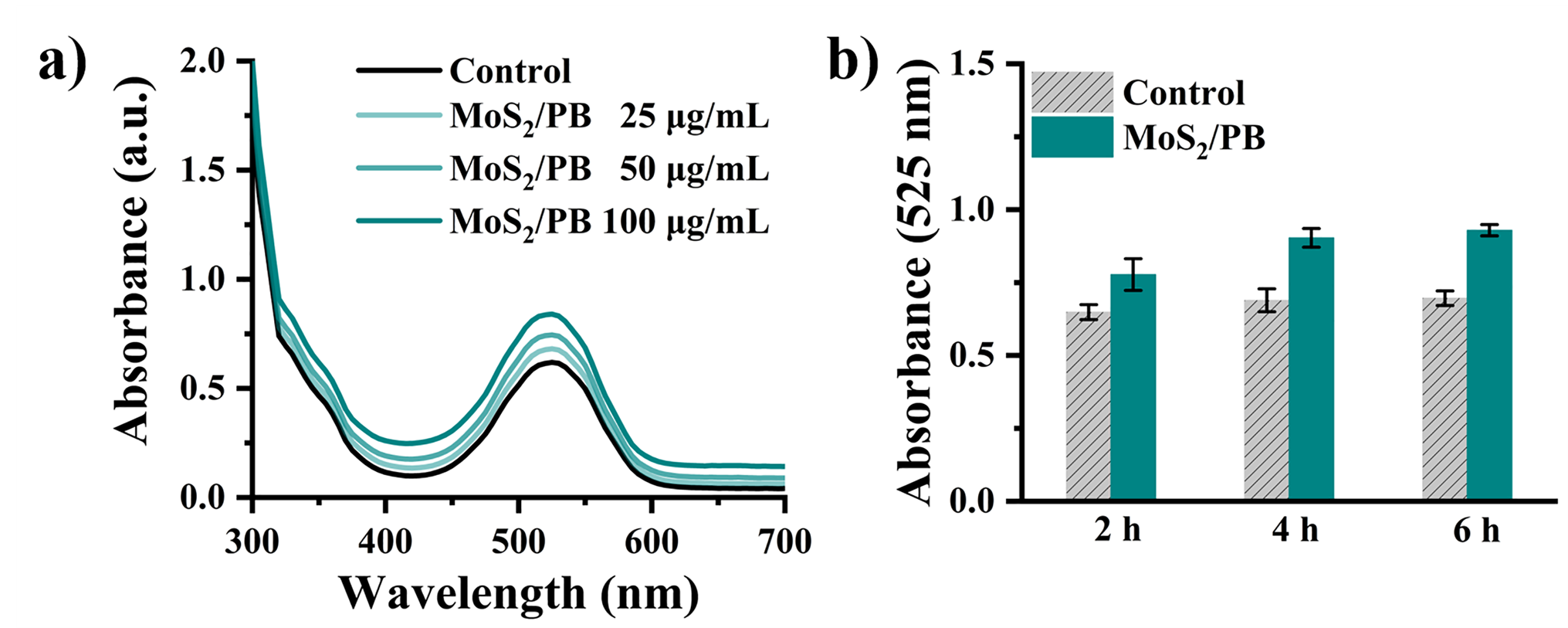


**Figure S30.** GSNOR activity of MoS_2_/PB. (a) Different concentrations. (b) Different time, n = 3.


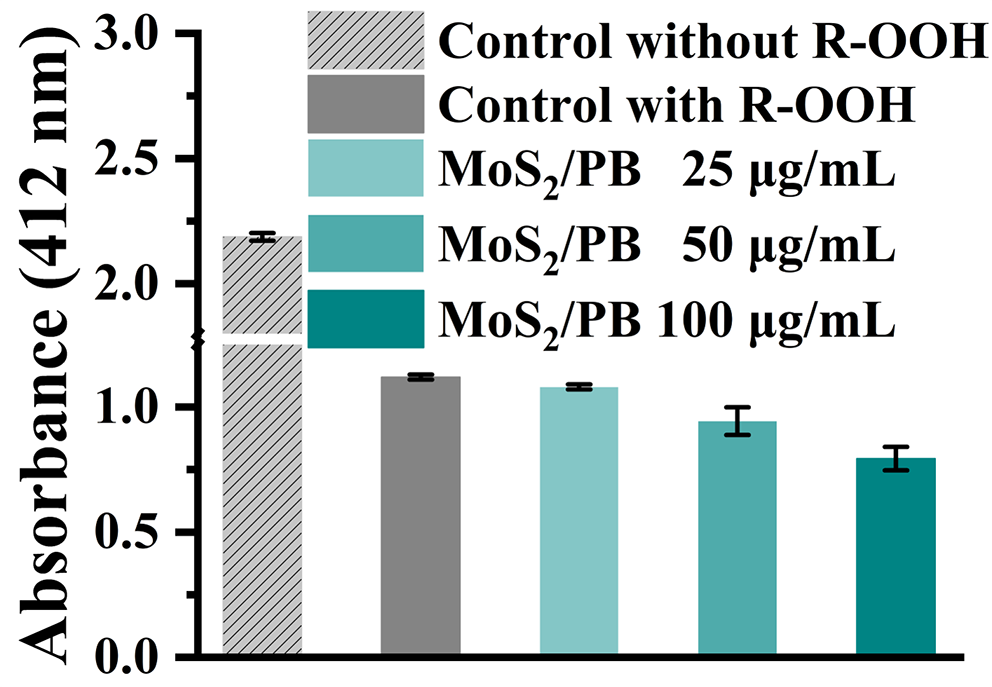


**Figure S31.** GSH-Px activity of MoS_2_/PB at different concentrations, n = 3.

1. **Supplemental Tables**

**Table S1.** The binding energy position of the 3d of Mo.

| Sample | Mo^4+^ 3d_5/2_ | Mo^4+^ 3d_5/2_ | Mo^4+^ 3d_3/2_ | Mo^4+^ 3d_3/2_ | Mo^6+^ 3d_3/2_ |
| --- | --- | --- | --- | --- | --- |
| PB | / | / | / | / | / |
| MoS_2_ | 227.8 eV | 229.2 eV | 230.9 eV | 232.3 eV | 234.4 eV |
| MoS_2_-CN^-^ | 228.0 eV | 229.1 eV | 231.0 eV | 232.2 eV | 234.5 eV |
| MoS_2_/ PB | 228.8 eV | 230.1 eV | 231.9 eV | 233.2 eV | 236.0 eV |

**Table S2.** The binding energy position of the 2p of S.

| Sample | 1-T S^2-^ 2p_3/2_ | 1-T S^2-^ 2p_1/2_ | 2-H S^2-^ 2p_3/2_ | 2-H S^2-^ 2p_1/2_ |
| --- | --- | --- | --- | --- |
| PB | / | / | / | / |
| MoS_2_ | 160.7 eV | 162.1 eV | 161.3 eV | 163.3 eV |
| MoS_2_-CN^-^ | 161.0 eV | 162.4 eV | 161.8 eV | 163.1 eV |
| MoS_2_/ PB | 161.2 eV | 162.9 eV | 161.9 eV | 164.1 eV |

**Table S3.** The binding energy position of the 2p of Fe.

| Sample | Fe^2+^ 2p_3/2_ | Fe^2+^ 2p_3/2_ | Fe^2+^ 2p_1/2_ | Fe^3+^ 2p_3/2_ | Fe^3+^ 2p_1/2_ |
| --- | --- | --- | --- | --- | --- |
| PB | 708.6 eV | 709.3 eV | 721.5 eV | 712.9 eV | 724.00 eV |
| MoS_2_ | / | / | / | / | / |
| MoS_2_-CN^-^ | 708.1 eV | / | 721.2 eV | / | / |
| MoS_2_/ PB | 708.5 eV | 709.1 eV | 721.3 eV | 712.5 eV | 723.8 eV |

**Table S4.** The percentage content of Mo^6+^ 3d_3/2_ in Mo by XPS.

| Sample | MoS_2_ | MoS_2_-HCl | MoS_2_-CN^-^ | MoS_2_/ PB |
| --- | --- | --- | --- | --- |
| Mo^6+^ 3d_3/2_ | 6% | 10% | 13% | 16% |

1. **Supplemental References**

[1] P. E. Blöchl, “Projector augmented-wave method” *Phys. Rev. B* **1994**, *50*, 17953–17979.

[2] S. Grimme, J. Antony, S. Ehrlich, et al., “A consistent and accurate *ab initio* parametrization of density functional dispersion correction (DFT-D) for the 94 elements H-Pu” *The Journal of Chemical Physics* **2010**, *132*, 154104.

[3] A. Lin, Q. Liu, Y. Zhang, et al., “A Dopamine-Enabled Universal Assay for Catalase and Catalase-Like Nanozymes” *Anal. Chem.* **2022**, *94*, 10636–10642.

[4] S. Wang, M. Cheng, S. Wang, et al., “A Self‐Catalytic NO/O_2_ Gas‐Releasing Nanozyme for Radiotherapy Sensitization through Vascular Normalization and Hypoxia Relief” *Advanced Materials* **2024**, 2403921.

[5] H. Dong, W. Du, J. Dong, et al., “Depletable peroxidase-like activity of Fe3O4 nanozymes accompanied with separate migration of electrons and iron ions” *Nat Commun* **2022**, *13*, 5365.

[6] Y. Wang, T. Li, H. Wei, et al., “Determination of the Maximum Velocity of a Peroxidase-like Nanozyme” *Anal. Chem.* **2023**, *95*, 10105–10109.

[7] K. Feng, Z. Wang, R. Guo, et al., “Exploring the Long‐term Catalytic Mechanism of Catalase‐Like Nanozymes Prepared by Flow Chemistry” *Adv Funct Materials* **2025**, e25623.

[8] K. Lu, H. He, J. Liu, et al., “Regional Electron Transfer and Band Regulation by Cluster‐Constrained Nanozyme Growth for Enhanced Catalytic Effect” *Small* **2025**, *21*, e08011.

[9] Y. Nosaka, A. Y. Nosaka, “Generation and Detection of Reactive Oxygen Species in Photocatalysis” *Chem. Rev.* **2017**, *117*, 11302–11336.

[10] J. Liu, X. Huang, F. Zhang, et al., “Metal-free multifunctional nanozymes mimicking endogenous antioxidant system for acute kidney injury alleviation” *Chemical Engineering Journal* **2023**, *477*, 147048.

[11] L. Yan, Z. Cao, L. Ren, et al., “A Sonoresponsive and NIR‐II‐Photoresponsive Nanozyme for Heterojunction‐Enhanced ‘Three‐in‐One’ Multimodal Oncotherapy” *Adv Healthcare Materials* **2024**, *13*, 2302190.

[12] B. J. G. Rousseau, A. V. Soudackov, R. R. Tuttle, et al., “Computational Insights into the Mechanism of Nitric Oxide Generation from *S* -Nitrosoglutathione Catalyzed by a Copper Metal–Organic Framework” *J. Am. Chem. Soc.* **2023**, *145*, 10285–10294.

[13] Y. Zhang, W. Liu, X. Wang, et al., “Nanozyme‐Enabled Treatment of Cardio‐ and Cerebrovascular Diseases” *Small* **2023**, *19*, 2204809.

[14] F. Zhang, M. Cheng, Z. Sun, et al., “Combined acid rain and lanthanum pollution and its potential ecological risk for nitrogen assimilation in soybean seedling roots” *Environmental Pollution* **2017**, *231*, 524–532.

[15] L. Wu, Y. Luo, C. Wang, et al., “Self-Driven Electron Transfer Biomimetic Enzymatic Catalysis of Bismuth-Doped PCN-222 MOF for Rapid Therapy of Bacteria-Infected Wounds” *ACS Nano* **2023**, *17*, 1448–1463.
